# Supplementary material for: Computational reproducibility of Jupyter notebooks from biomedical publications
Source: Gigascience. 2024 Jan 11;13:giad113. doi: 10.1093/gigascience/giad113 (PMC10783158; doi:10.1093/gigascience/giad113)
Supplement: giad113_GIGA-D-22-00259_Original_Submission [file giad113_giga-d-22-00259_original_submission.pdf]

GigaScience

Computational reproducibility of Jupyter notebooks from biomedical publications

--Manuscript Draft--

**Manuscript Number:**	GIGA-D-22-00259
**Full Title:**	Computational reproducibility of Jupyter notebooks from biomedical publications
**Article Type:**	Research
**Funding Information:**	Carl-Zeiss-Stiftung — Dr. Sheeba Samuel<br>Alfred P. Sloan Foundation (G-2021-17106) — Dr. Daniel Mietchen<br>Deutsche Forschungsgemeinschaft (INST 275/334-1 FUGG) — Not applicable<br>Deutsche Forschungsgemeinschaft (INST 275/363-1 FUGG) — Not applicable
**Abstract:**	Background<br>Jupyter notebooks allow to bundle executable code with its documentation and output in one interactive environment, and they represent a popular mechanism to document and share computational workflows, including for research publications.<br>Results<br>Here, we analyze the computational reproducibility of 9625 Jupyter notebooks from 1117 GitHub repositories associated with 1419 publications indexed in the biomedical literature repository PubMed Central. 8160 of these were written in Python, including 4169 that had their dependencies declared in standard requirement files and that we attempted to re-run automatically. For 2684 of these, all declared dependencies could be installed successfully, and we re-ran them to assess reproducibility. Of these, 396 notebooks ran through without any errors, including 245 that produced results identical to those reported in the original. Running the other notebooks resulted in exceptions.<br><br>Conclusions<br>We zoom in on common problems and practices, highlight trends and discuss potential improvements to<br><br>Jupyter-related workflows associated with biomedical publications.
**Corresponding Author:**	Daniel Mietchen<br>Ronin Institute for Independent Scholarship<br>Montclair, New Jersey UNITED STATES
**Corresponding Author Secondary Information:**	
**Corresponding Author's Institution:**	Ronin Institute for Independent Scholarship
**Corresponding Author's Secondary Institution:**	
**First Author:**	Sheeba Samuel
**First Author Secondary Information:**	
**Order of Authors:**	Sheeba Samuel<br>Daniel Mietchen
**Order of Authors Secondary Information:**	
**Additional Information:**	
**Question**	**Response**
Are you submitting this manuscript to a special series or article collection?	No

Powered by Editorial Manager® and ProduXion Manager® from Aries Systems Corporation

**Experimental design and statistics**<br><br>Full details of the experimental design and statistical methods used should be given in the Methods section, as detailed in our Minimum Standards Reporting Checklist. Information essential to interpreting the data presented should be made available in the figure legends.<br><br>Have you included all the information requested in your manuscript?	Yes
**Resources**<br><br>A description of all resources used, including antibodies, cell lines, animals and software tools, with enough information to allow them to be uniquely identified, should be included in the Methods section. Authors are strongly encouraged to cite Research Resource Identifiers (RRIDs) for antibodies, model organisms and tools, where possible.<br><br>Have you included the information requested as detailed in our Minimum Standards Reporting Checklist?	Yes
**Availability of data and materials**<br><br>All datasets and code on which the conclusions of the paper rely must be either included in your submission or deposited in publicly available repositories (where available and ethically appropriate), referencing such data using a unique identifier in the references and in the "Availability of Data and Materials" section of your manuscript.<br><br>Have you have met the above requirement as detailed in our Minimum Standards Reporting Checklist?	Yes

Powered by Editorial Manager® and ProduXion Manager® from Aries Systems Corporation

Manuscript
Click here to access/download;Manuscript;Computational-reproducibility-of-Jupyter-notebooks.pdf

OXFORD (GIGA)n SCIENCE

GigaScience, 2017, 1–15

doi: xx.xxxx/xxxx
Manuscript in Preparation
Paper

PAPER

Computational reproducibility of Jupyter notebooks from biomedical publications

Sheeba Samuel[1,2*†] and Daniel Mietchen[3,4,5†‡]

[1]Heinz-Nixdorf Chair for Distributed Information Systems, Friedrich Schiller University Jena, Germany and [2]Michael Stifel Center Jena, Germany and [3]Ronin Institute, Montclair, New Jersey, United States and [4]Institute for Globally Distributed Open Research and Education (IGDORE) and [5]FIZ Karlsruhe — Leibniz Institute for Information Infrastructure, Berlin, Germany

[*]sheeba.samuel@uni-jena.de
[†]These authors contributed equally to this work
[‡]daniel.mietchen@ronininstitute.org

Abstract

Background Jupyter notebooks allow to bundle executable code with its documentation and output in one interactive environment, and they represent a popular mechanism to document and share computational workflows, including for research publications.
Results Here, we analyze the computational reproducibility of 9625 Jupyter notebooks from 1117 GitHub repositories associated with 1419 publications indexed in the biomedical literature repository PubMed Central. 8160 of these were written in Python, including 4169 that had their dependencies declared in standard requirement files and that we attempted to re-run automatically. For 2684 of these, all declared dependencies could be installed successfully, and we re-ran them to assess reproducibility. Of these, 396 notebooks ran through without any errors, including 245 that produced results identical to those reported in the original. Running the other notebooks resulted in exceptions.
Conclusions We zoom in on common problems and practices, highlight trends and discuss potential improvements to Jupyter-related workflows associated with biomedical publications.

Key words: Computational reproducibility; Jupyter notebooks; PubMed Central; GitHub; dependency decay; Python

Introduction

Many factors contribute to the progress of scientific research, including the precision, scale, and speed at which research can be performed and shared and the degree to which research processes and their outcomes can be trusted [1, 2]. This trust, in turn, and the credibility that comes with it, are a social construct that depends on past experience or proxies to it [3, 4, 5]. A good proxy here is reproducibility, at least in principle [6]: if a study addressing a particular research question can be re-analyzed and that analysis leads to the same conclusions as the original study, then these conclusions can generally be more trusted than if the conclusions differ between the original and the replication study.

Reproducibility issues in contemporary research

Over recent years, the practical replicability of published research has come into focus and turned into a research area in and of itself [7, 8]. As a result, systematic issues with reproducibility have been the subject of many publications in various research fields as well as prominent mentions in the mass media [9]. These research fields range from psychology [10] to cell culture [11, 12] to ecology [13], geosciences [14] and beyond and include software-affine domains such as health informatics [15], human-computer interactions [16], artificial intelligence [17], software engineering [18] and research software [19]. This is often framed in terms of a "reproducibility crisis" [20], though that may not necessarily be the most productive approach to addressing the underlying issues [21, 22, 23]. In more practical terms, Näpflin et al. [24] observe that "appropriate

Compiled on: October 5, 2022.
Draft manuscript prepared by the author.

Key Points

- We present a systematic attempt of automatically re-running Jupyter notebooks underlying research reported in articles indexed in PubMed Central.
- The large majority of these notebooks could not be executed automatically, mostly due to issues with the documentation of dependencies.
- The manuscript peer review process often does not properly address the review of associated notebooks.

workflow documentation is essential".

Terminology

Within this broader context, distinctions between replicability, reproducibility, and repeatability are often important or even necessary [25] but not consistently made in the literature [26]. A potential solution to this confusion is the proposed distinction [27] between *Methods reproducibility* (providing enough detail about the original study that the procedures and data can be repeated exactly), *Results reproducibility* (obtaining the same results when matching the original procedures and data as closely as possible) and *Inferential reproducibility* (leading to the same scientific conclusions as the original study, either by reanalysis or by independent replication).

In the following, we will concentrate on "Methods reproducibility in computational research", i.e. using the same code on the same data source. For this, we will use the shorthand "Computational reproducibility". In doing so, we are conscious that the "same code" can yield different results depending on the execution environment and that the "same data source" might actually mean different data if the data source is dynamic or if the code involves manipulating the data in a way that changes over time. We are also aware that the shorthand "Computational reproducibility" can also be applied, e.g., to "Results reproducibility in computational research" in cases where the algorithm described for the original study was re-implemented in a follow-up study. For instance, Burlingame et al. [28] were striving for *Results reproducibility* when they re-implemented the PhenoGraph algorithm – which originally only ran on CPUs – such that it could be run on GPUs and thus at higher speed. However, *Results reproducibility* is not the focus of our study.

Computational reproducibility in biomedical research

In light of the reproducibility issues outlined above, there have been calls for better standardization of biomedical research software – see Russell et al. [29] for an example. In line with such standardization calls, a number of guidelines or principles to achieve methods reproducibility in several computational research contexts have been proposed. For instance, [30], [31] and [32] laid out principles for reproducible computational research in general. In a similar vein, [33] and [34] looked at specifics of computational reproducibility in the life sciences, [35] explored the use of Docker – a containerization tool – in reproducibility contexts, and [36] looked at the reproducibility of R scripts archived in an institutional repository, while [37], [38] as well as [39, 40] and [41] zoomed in on Jupyter notebooks, a popular file format for documenting and sharing code. While most of these are language agnostic, language-specific approaches to computational reproducibility have also been outlined, e.g. for Python [42].

However, compliance with such standards and guidelines is not a given [29, 43, 44], so we set out to measure it specifically for Jupyter notebooks in the life sciences and to explore options to bridge the gap between recommended and actual practice. In order to do so, we mined a popular repository of biomedical fulltexts (PubMed Central) for mentions of Jupyter notebooks alongside mentions of a popular repository for open-source software (GitHub).

PubMed Central

PubMed Central (PMC)[1] is a literature repository containing full texts of biomedical articles. At the time of writing, it contained about 7.5 million articles. Founded in the context of the Open Access mandate issued by the National Institutes of Health (NIH) in the United States [45], PMC is operated by the National Center for Biotechnology Information (NCBI), a branch of the National Library of Medicine (NLM), which is part of the NIH. PMC hosts the articles using the Journal Article Tagging Suite (JATS), an XML standard, and makes them available for manual and programmatic access in various ways, of which we used the Entrez API [46].

GitHub

GitHub[2] is a website that combines git-based version control with support for collaboration and automation. It is a popular place for sharing software and developing it collaboratively, including for Jupyter notebooks [43] and for code associated with research articles available through PubMed Central [29].

Jupyter

Jupyter notebooks[3] [47, 48] are a computing environment in which code, code documentation, and output of the code can be explored interactively. They have become a popular mechanism to share computational workflows in a variety of fields [47], including astronomy [49, 50] and biosciences [51]. Here, we build on past studies of the reproducibility of Jupyter notebooks [43, 38] and analyze Jupyter notebooks available through GitHub repositories associated with publications available through the biomedical literature repository PubMed Central.

Jupyter and reproducibility

Jupyter notebooks can, in principle, be used to enhance reproducibility, and they are often presented as such, yet using them does not automatically confer reproducibility to the code they contain. Several studies have been conducted in recent years to explore the reproducibility of Jupyter Notebooks. A recent one has investigated the reproducibility of Jupyter notebooks associated with five publications from the PubMed Central database [51]. In their reproducibility analysis, they looked for the presence of notebooks, source code artifacts, documentation of the software requirements, and whether the notebooks can be re-executed with the same results. According to their results, the authors successfully reproduced only three of 22 notebooks from five publications. Rule et al. [43] explored 1 million notebooks available on GitHub. In their

1 https://www.ncbi.nlm.nih.gov/pmc/
2 https://github.com/
3 https://jupyter.org/

study, they explored repositories, language, packages, notebook length, and execution order, focusing on on the structure and formatting of computational notebooks. As a result, they provided ten best practices to follow when writing and sharing computational analyses in Jupyter Notebooks [37]. Another study [44] focused on the reproducibility of 1.4 million notebooks collected from GitHub. It provides an extensive analysis of the factors that impact reproducibility based on Jupyter notebooks. Chattopadhyay et al. [52] reported on the results of a survey conducted among 156 data scientists on the difficulties when working with notebooks. Other studies focus on best practices on writing and sharing Jupyter notebooks [37, 44, 40, 41]. As a result, tools have been developed to support provenance and reproducibility in Jupyter Notebooks [53, 54, 55, 56]. Cases where Jupyter notebooks have played a key role in some actual replication attempts have also begun to appear in the literature. For instance, Baker et al. [57] assembled a Jupyter notebook as part of a published correction. Shortly after we had created our corpus, a paper was published with a Jupyter notebook that enabled others to reproduce the computational workflows, ultimately leading to the retraction of the original work, as detailed in Meyerowitz-Katz et al. [58].

Wikidata

Wikidata is a cross-disciplinary and multilingual database through which a global community curates FAIR and open data to serve as general reference information [59, 60]. This includes information about key elements of the research ecosystem, from researchers to research fields and research organizations, from methods to datasets, software and publications. This information can then be explored in various ways, e.g. through the visualization tool Scholia [61], which provides profiles for different types of entities or relationships. For entities of the type Jupyter notebook (known to Wikidata as Q70357595), the most relevant profile types are those for a topic[4], a software [5] or a resource used[6].

Environmental footprint

Computations ultimately require physical resources, and both the production and the use of these resources can have a considerable environmental footprint [62]. The more reproducible some workflows become, the more accurate their environmental footprint can be assessed [63]. This can then lead to an optimization of the environmental footprint, especially since it often correlates with the financial footprint of using computational resources [64]. One of our aims in this study is thus to get an overview of the contribution of Jupyter-based workflows to the environmental footprint of biomedical research involving computation. This is in line with the recommendation in Lannelongue et al. [65] to integrate routine environmental footprint assessment into research practice.

Methods

Pipeline

In this section, we describe the key steps of the pipeline we used for assessing the reproducibility of Jupyter notebooks (RRID:SCR_018315) associated with publications extracted from PubMed Central (RRID:SCR_004166). The driver file for running the workflow is documented in r0_main.py and the driver notebook for the analysis of the collected data is documented in the notebook named "Index.ipynb". Figure 1 provides an overview of the workflow used in this study.

We used the *esearch* function to search PMC for Jupyter notebooks on 24th February, 2021. We looked for publications that mentioned GitHub together with either the string "Jupyter" or some closely associated ones, namely "ipynb" (the file ending/extension of Jupyter notebooks) or "iPython" (the name of a precursor to Jupyter). The search query used was "(ipynb OR jupyter OR ipython) AND github". Based on the primary PMC IDs received from the *esearch* utility, we retrieved records in the XML format using the *efetch* function and collected the publication metadata from PMC [45] using NCBI Entrez utilities via Biopython [66].

In the next step, we processed the XML fetched from PMC. We used an SQLite database[7] for storing all the data related to our pipeline. We collected information on journals and articles. We first extracted information about the journal. For this, we created a database table for the journal and extracted the ISSN[8] (International Identifier for serials), the journal title, the NLM's (National Library of Medicine) abbreviated journal title, and the ISO[9] (International Organization for Standardization) abbreviation.

We then created a database table for the articles and populated it with article metadata. The metadata includes the article name, Pubmed ID, PMC ID, Publisher id and name, DOI, subject, the dates when the article was received, accepted, and published, the license, the copyright statement, keywords, and the GitHub repositories mentioned in the publication. For each article, we also extracted the Medical Subject Headings (MeSH terms)[10] to get the subject area of the article.

To extract the GitHub repositories mentioned in each article, we looked for mentions of GitHub links anywhere in the article, including the abstract, the article body, data availability statement, and supplementary information. GitHub links were available in different formats. We normalized them to the standard format 'https://github.com/{username}/{repositoryname}'. For example, we extracted the GitHub repository from nbviewer[11] links and transformed its representation to the standard format. We excluded 692 GitHub links that mentioned only the username or organization name or github pages and not a specific repository name. After preprocessing and extracting GitHub links from each article, we added the GitHub repositories to the database table for the corresponding articles. Likewise, we linked the article's entry in the table to the journal where it was published. We also collected information on the authors of the article in a separate database table: we created an author database table, extracted the first and last name, ORCID, email, and connected these data to the corresponding entries in the article table.

Based on the GitHub repository name collected from the article, we checked whether these repositories were available at the original link or not. If the repository existed, we cloned it (ignoring branches, i.e. just taking the base one, which is usually called "main") and collected information about the repositories using the GitHub REST API[12]. On that basis, we created a repository database table. For each GitHub repository, an entry is created in the table and connected to the article where it is mentioned. We collected the execution environment information by looking into the dependency information declared in the repositories in terms of files like *requirements.txt*, *setup.py* and *pipfile*. Additional information for each repository is also collected from the GitHub API. This includes the dates of the creation, updates, or pushes to the repository, and the programming languages used in each repository. Further information includes the number of subscribers, forks, issues, downloads,

4 https://scholia.toolforge.org/**topic**/Q70357595
5 https://scholia.toolforge.org/**software**/Q70357595
6 https://scholia.toolforge.org/**use**/Q70357595

7 https://www.sqlite.org
8 https://www.issn.org/
9 https://www.iso.org
10 https://www.ncbi.nlm.nih.gov/mesh
11 https://nbviewer.org/
12 https://docs.github.com/en/rest/guides/getting-started-with-the-rest-api

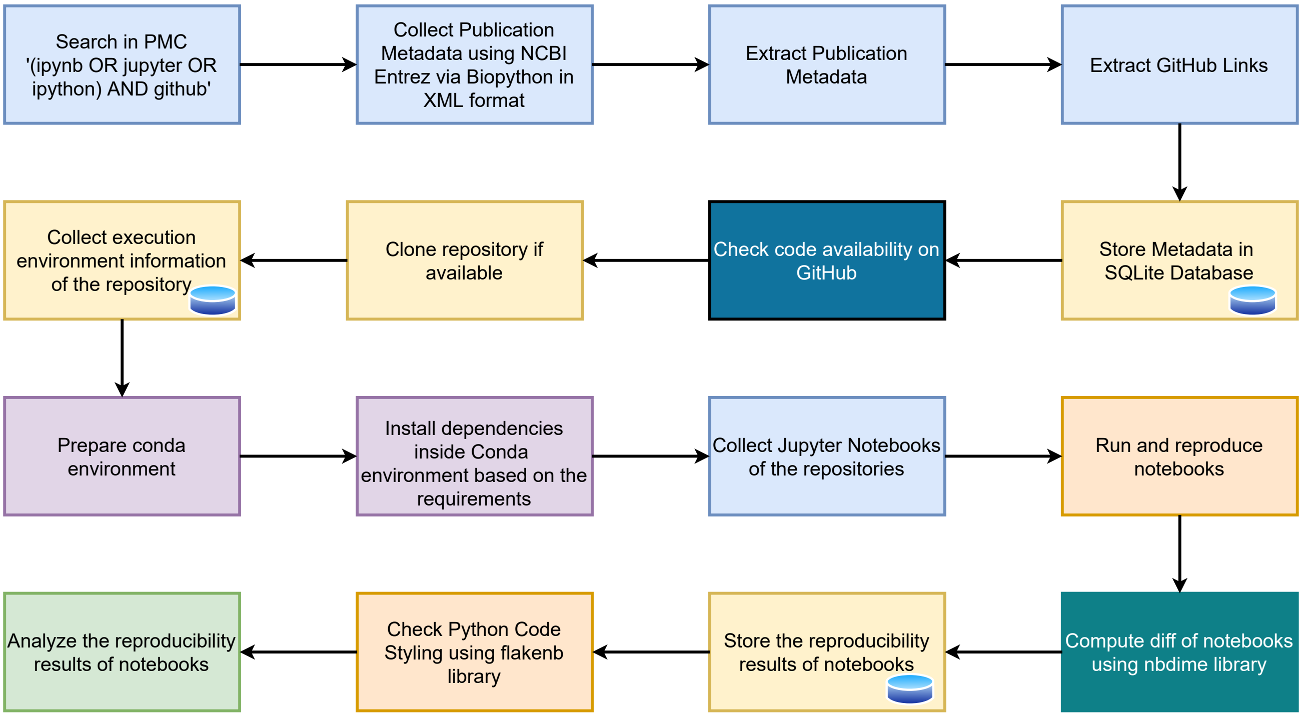

Figure 1. Fully automated workflow used for assessing the reproducibility of Jupyter notebooks from publications indexed in PubMed Central: the PMC search query resulted in a list of article identifiers that were then used to retrieve the full-text XML, from which publication metadata and GitHub links were extracted and entered into an SQLite database. If the links pointed to valid GitHub (RRID:SCR_002630) repositories containing valid Jupyter notebooks, then metadata about these were gathered, and the Python-based notebooks were run with all identifiable dependencies, and their results analyzed with respect to the originally reported ones.

license name and type, total releases, and total commits after the respective dates for when the article was published, accepted, and received. After collecting and creating these data tables, we ran a pipeline to collect the Jupyter notebooks contained in the GitHub repositories. The code for the pipeline is adapted from [38, 67]. Hence, the method to reproduce the notebooks in this study is similar to [38]. For each notebook, we collected information on the name, nbformat, kernel, language, number of different types of cells, and the maximum execution count number. We extracted the source and output of each cell for further analysis. Using Python Abstract Syntax Tree (AST)[13] the pipeline extracted information on the use of modules, functions, classes, and imports.

After collecting all the required information for the execution of Python notebooks from the repositories, we prepared a Conda[14] environment based on the python version declared in the notebook. Conda is an open source package and environment management system which helps users to easily find and install packages and create, save, load and switch between environments. The pipeline then installed all the dependencies collected from the corresponding files like *requirements.txt*, *setup.py* and *pipfile* inside the Conda environment. For the repositories that did not provide any dependencies using the above mentioned files, the pipeline executed the notebooks by installing all the anaconda dependencies[15]. Anaconda is a Python and R distribution which provides data science packages including *scikit-learn*, *numpy*, *matplotlib*, and *pandas*.

We used the nbdime[16] library from Project Jupyter to compute diffs of the notebooks. We used the tools adapted from [38, 67]. The code from [38] provides a basis for reproducing Jupyter notebooks from GitHub repositories. The ReproduceMeGit [67] extended from [38], is a visualization tool for analyzing the reproducibility of Jupyter Notebooks, along with provenance information of the execution. ReproduceMeGit provides the difference between the results of the executions of notebooks using the nbdime library. These two tools provide the basis for our code for the reproducibility study.

After collecting the notebooks, we also ran a Python code styling check using the flakenb[17] library on the notebooks, since code styling consistency is a potential indicator for the extent of care that went into a given piece of software. The flakenb library is a tool for code style guide enforcement for notebooks. It helps to check code against some of the style conventions in PEP 8[18], a style guide for Python code. The flakenb library provides an *ignore* flag to ignore some specified errors. In this study, we did not use this flag and collected all errors detected by the library. For the styling of notebooks, we collected information on the pycode styling error code and description[19].

Reproduction

The complete pipeline was run on the Friedrich Schiller University Ara Cluster[20]. The computational experiments were performed on a Skylake Standard Node (2x Intel Xeon Gold 6140 18 Core 2,3 GHz, 192 GB RAM). This node has two CPUs, each with 18 cores, and 192 GB RAM in total. The complete pipeline ran in 117 hours and 52 minutes from 24th–28th February 2021. We then used the website https://green-algorithms.org v2.2 [62] to estimate that the pipeline run drew 47.38 kWh. Based in Germany, this has a carbon footprint of 16.05 kg CO_2e, which is equivalent to 17.51 tree-months.

13 https://docs.python.org/3/library/ast.html
14 https://docs.conda.io/en/latest/
15 https://docs.anaconda.com/anaconda/packages/pkg-docs/
16 https://github.com/jupyter/nbdime
17 https://github.com/s-weigand/flake8-nb
18 https://www.python.org/dev/peps/pep-0008/
19 https://pycodestyle.pycqa.org/en/latest/intro.html
20 https://wiki.uni-jena.de/pages/viewpage.action?pageId=22453005

Results

In this section, we present the results of our study on analyzing the computational reproducibility of Jupyter notebooks from biomedical publications. We extracted metadata from 1419 publications from PubMed Central. These articles had been published in 373 journals and had 2398 mentions of GitHub repository links. At the time of data collection, 49 GitHub repositories mentioned in the articles were not accessible, returning a "page not found" error instead. Out of 2177 unique and valid GitHub repositories cloned, only 1117 had one or more Jupyter notebooks. From these repositories, a total of 9625 Jupyter notebooks were downloaded for further reproducibility analysis.

General statistics of our study

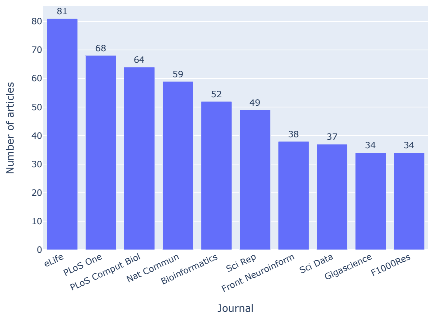

Figure 2. Journals with the highest number of articles that had a valid GitHub repository and at least one Jupyter notebook. In the figures, journal names are styled as in the XML files we parsed, e.g. ("PLoS Comput Biol"). In the text, we use the full name in its current styling, e.g. "PLOS Computational Biology".

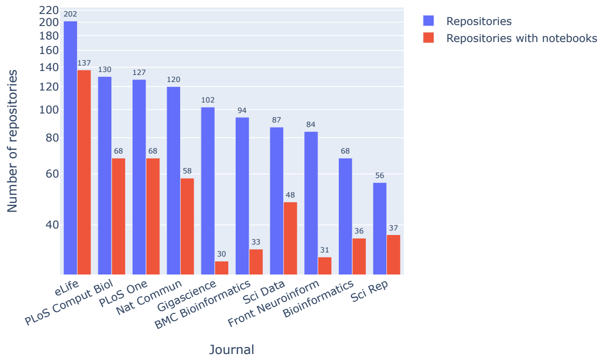

Figure 3. Journals by the number of GitHub repositories and by the number of GitHub repositories with at least one Jupyter notebook.

Figure 2 shows the top ten journals with the highest number of articles that had a valid GitHub repository with at least one Jupyter notebook. Figure 3 shows the journals by the number of GitHub repositories and repositories with Jupyter notebooks. It was followed by *PLOS ONE* and *PLOS Computational Biology*. The ratio of notebooks per GitHub repository varies across journals, with the range being between 3.4:1 in *GigaScience*, 2:1 in *Nature Communications*, and 1.5:1 in *Scientific Reports*. From the 1117 repositories with Jupyter notebooks, 290 (25.9%) of repositories had one Jupyter notebook, 462 (41.4%) had two notebooks, and 249 (22.3%) had ten or more notebooks. 6,782 (70.4%) of the notebooks belonged to repositories with ten or more notebooks.

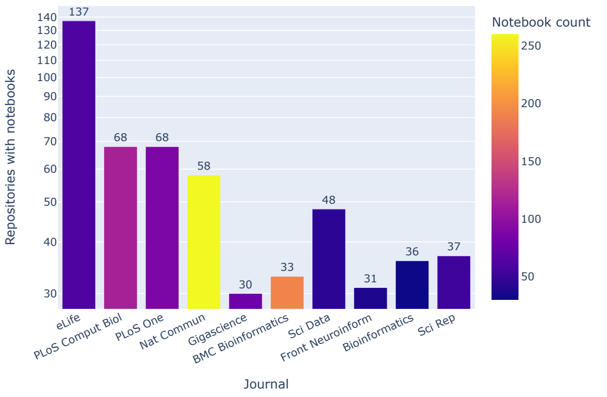

Figure 4. Journals by number of GitHub repositories with Jupyter notebooks. For each journal, the notebook count gives the maximum number of notebooks within a repository associated with an article published in the journal.

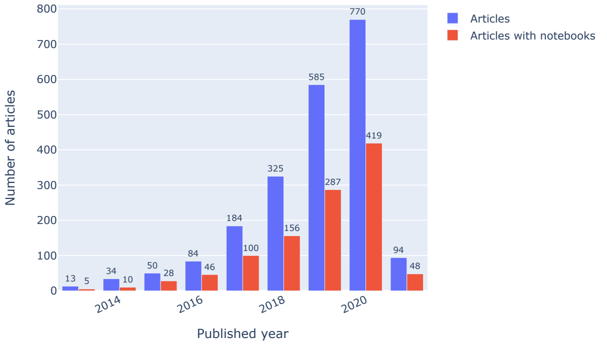

Figure 5. Articles by number of GitHub repositories with at least one Jupyter notebook by year.

Figure 5 shows the maximum number of notebooks for articles published in the respective journal. Among the top ten journals with notebooks, *Nature Communications* had the maximum number of notebooks; however, it ranked fourth in terms of journals with repositories with notebooks. Figure 5 shows the timeline of the articles by the number of Github repositories with at least one Jupyter notebook. This indicates a growing trend of articles with notebooks.

In parallel to exploring trends related to Jupyter notebooks, we analyzed the uptake of ORCID identifiers[21] over time in the collected journal articles with notebooks (Figure 6). ORCID provides a persistent digital identifier to uniquely identify authors and contributors of scholarly articles. While iPython notebooks go back to 2001, the Jupyter notebooks with kernels for multiple languages became available in 2014, whereas ORCID was launched in 2012. Hence, both are relatively recent innovations in the scholarly communi-

21 https://orcid.org/

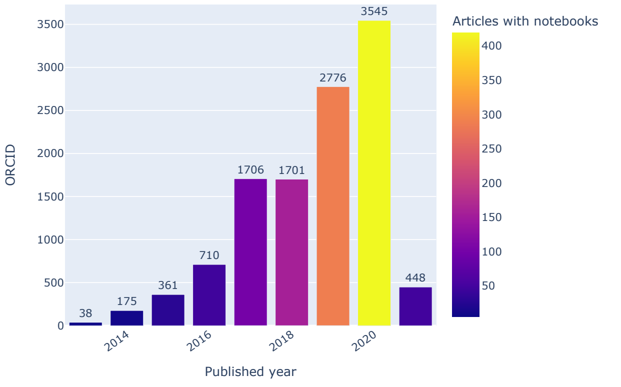

Figure 6. ORCID usage in our collection. Bars indicate the total number of ORCIDs found each year for authors of articles in our collection. Colors indicate the number of articles that year with Jupyter notebooks. Note that data for 2021 is incomplete, as only articles published by mid-February have been included.

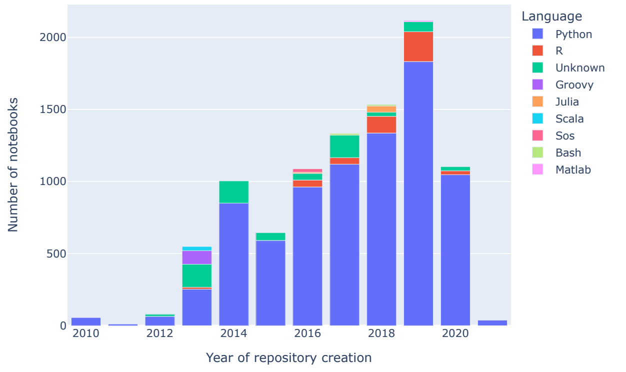

Figure 8. Relative proportion of the most frequent programming languages used in the notebooks per year.

cations ecosystem, and their respective uptake processes occur in parallel.

There are in total 11594 authors in the 1419 publications. We have not performed any author disambiguation to distinguish unique authors in our corpus. However, such disambiguation is taking place at scale in Wikidata (see Discussion). There are 2,720 (23.46%) authors with ORCID and 8,874 (76.54%) without. In 2020, there were 3545 mentions of author ORCIDs and the highest number of articles (419) with notebooks. The figure shows an increase in the usage of ORCID as well as articles with notebooks from 2016.

Programming languages

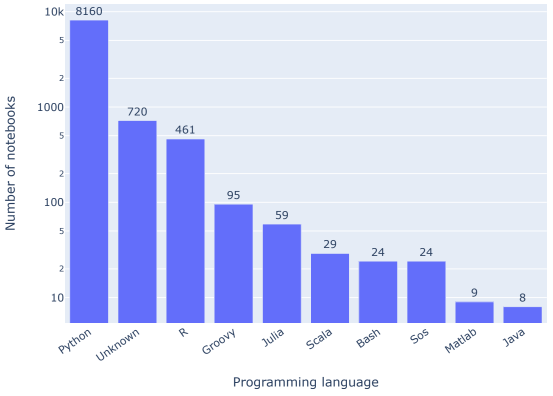

Figure 7. Programming languages of the notebooks. "Unknown" means the language kernel used was not indicated in a standardized fashion.

Figure 7 and Figure 8 show analyses of the programming languages used in the Jupyter notebooks present in the collected publications. Figure 7 presents (using a log scale) the most common programming languages used in the notebooks. Python (84.8%) is the most common programming language, followed by unknown (7.5%) and R (4.8%). Unknown notebooks are those which do not declare the programming language or its version in the notebook. A total of 720 notebooks do not declare a programming language. From the figure, we can see that the Jupyter ecosystem is not just Python anymore, but Python is most prominent, and none of the other languages have overtaken the "Unknown" group, which is primarily due to early notebooks in which Python was hardcoded, or the language stated in some other non-standard fashion. Jupyter Notebooks are also used for other languages like Bash, Matlab, and Java. Figure 8 shows the top programming languages used in notebooks based on the published year of the article. There is a steady use of Python in Jupyter Notebooks. However, fewer notebooks have undeclared programming language in 2018 and 2020. There is also an increase in the use of R in notebooks. Authors can see the timeline of other programming languages in the analysis notebook provided in the repository.

Versions

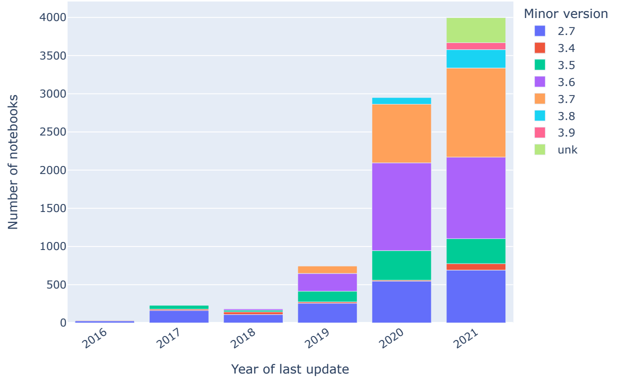

Figure 9. Python notebooks by minor Python version by year of last commit to the GitHub repository.

Figure 9 shows the Python version of notebooks based on the year in which the repository was last updated. 2471 notebooks have Python version 3.6, followed by 2031 notebooks with Python version 3.7. Python version 3.6 and 3.7 are commonly used in recent years, followed by version 2.7. There are also some python notebooks without any version declared. 6028 notebooks have Python major version 3, 1802 notebooks have Python major version 2, and 329 notebooks have an unknown Python version.

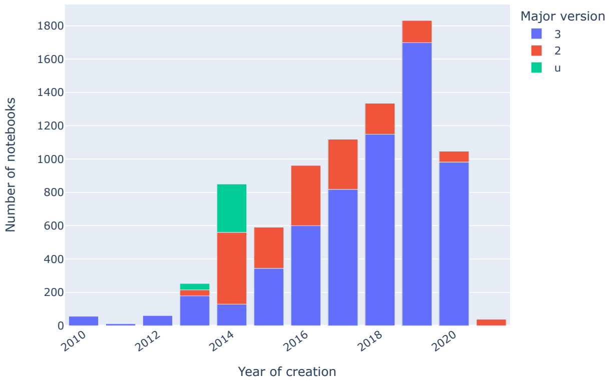

Figure 10. Python notebooks by major Python version by year of first commit.

Notebook structure

Figure 11 shows the statistics on the structure of notebooks. Notebooks have a median of 20 cells and 13 code cells. The average number of cells with outputs in notebooks found in our study is three, with zero being the least (Figure 11b). The maximum number of cells, code cells, and cells with output seen in a notebook are 95, 431, and 163, respectively. The maximum number of raw and empty cells seen in a notebook is 49 and 31, respectively. Raw cells let the users write output directly and the kernel does not evaluate them. The average number of markdown cells in notebooks is six, with the maximum being 383. 6311 (65.77%) of the notebooks have markdown cells, while 3284 (34.23%) notebooks do not. 96.58% of notebooks use English in the markdown cells; While 46.27% notebooks use only English in the markdown cells. In addition to English, French (11.76%) and Danish (3.96%) are the other popular natural languages used in the markdown. In 1909 (30.25%) notebooks, we could not detect the language in the markdown cells. Further analysis of markdown cells shows that the average number of lines and words seen in markdown cells are 20 and 145, respectively. Paragraphs and headers, the most commonly seen markdown elements, appear in 92.65% and 81.81% notebooks, respectively. 1,449 (17.76%) notebooks do not have execution numbers and 6,710 (82.24%) notebooks have execution numbers. The maximum execution count seen in a notebook is 2076 (Figure 11d).

Notebook naming

Figure 12 shows the most frequently used titles in notebooks from our collected data. "Untitled", "programming" and "index" are the three most common notebook names. There are 63 (0.65%) whose title is or starts with "Untitled". There are 21 (0.22%) notebooks that contain the name 'Copy'. We also see many notebooks with the string 'test' in their names (Figure 13). 1,070 (11.12%) notebooks have names that are not recommended by the POSIX fully portable filenames guide [38]. Only four notebooks have names that are disallowed in Windows. There are no notebooks without a title (i.e., notebooks with just a '.ipynb' extension). Figure 14 shows the distribution of length of notebook title. The average length of the notebook title is 18 characters, with a maximum of 123 characters and a minimum of 2.

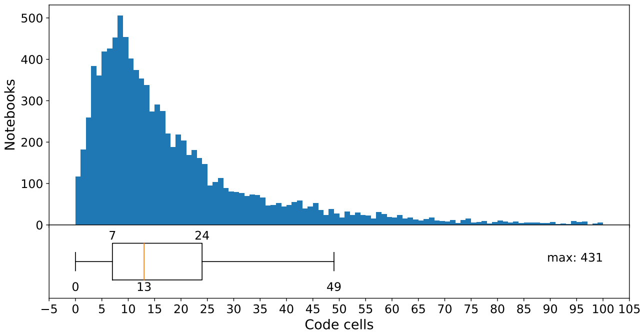

(a) Distribution of the number of code cells across notebooks in our corpus.

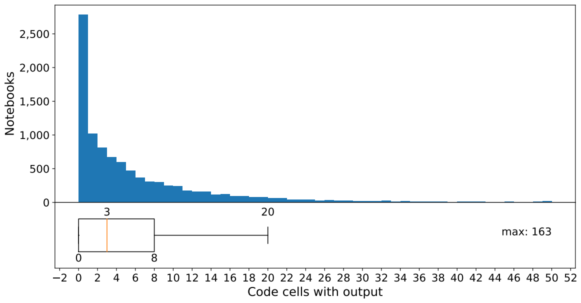

(b) Distribution of the number of code cells with outputs across notebooks in our corpus.

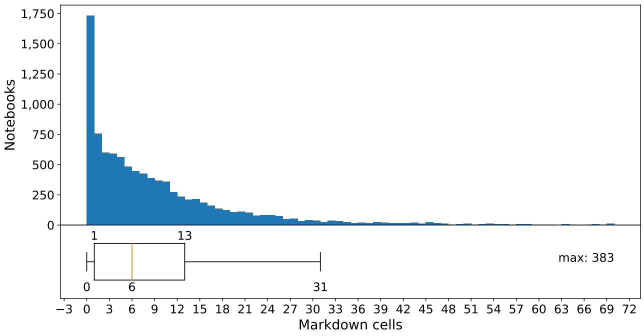

(c) Distribution of the number of Markdown cells across notebooks in our corpus.

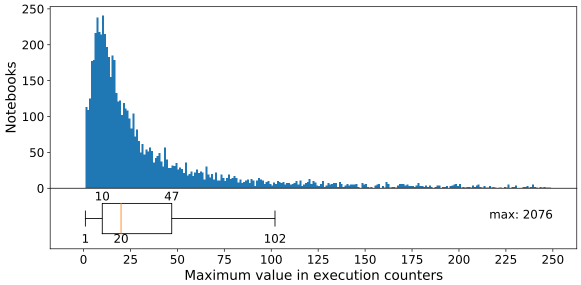

(d) Distribution of the maximum execution count across notebooks in our corpus.

Figure 11. Analysis of the notebook structure

Notebook modules

Figure 15 shows the analysis of modules declared in notebooks. Using AST[22], we analyzed the valid Python notebooks. 5,248 (69.06%)

22 https://docs.python.org/3/library/ast.html

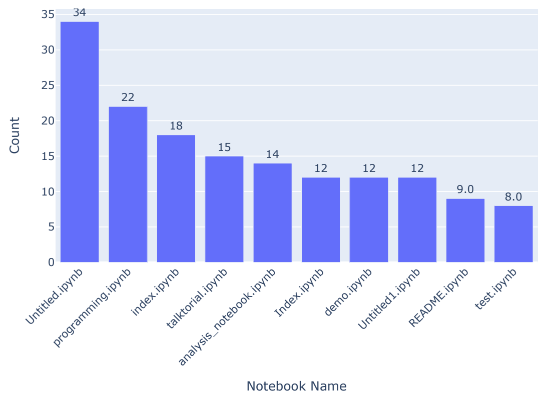

Figure 12. Most frequent notebook titles.

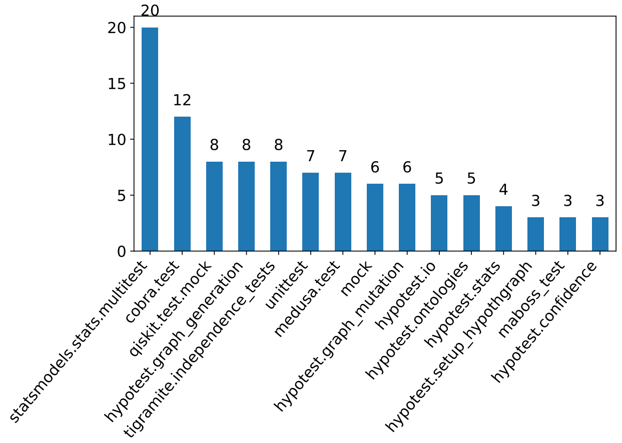

Figure 13. Notebooks with the string `test`

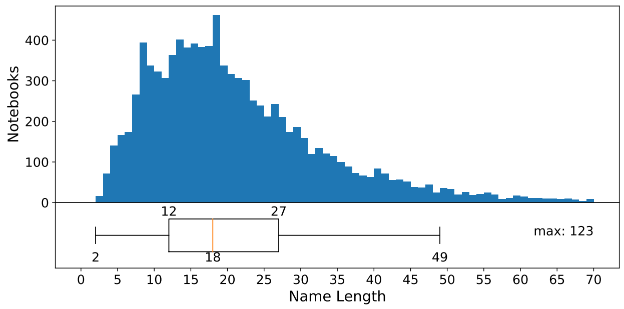

Figure 14. Notebook title length

notebooks had imports, of which 714 (9.40%) had local imports, while 5,216 (68.64%) had external modules. Local imports denote the import of modules defined in the notebook repository's directory. There are 1035 local and 38229 external modules declared in the collected Python notebooks. Figure 15 shows the top ten commonly used Python modules declared in the notebooks. The most used modules are *numpy* (3255), *pandas* (2428), and *matplotlib.pyplot* (2411). These are widely used modules for data manipulation, analytics, and visualizations.

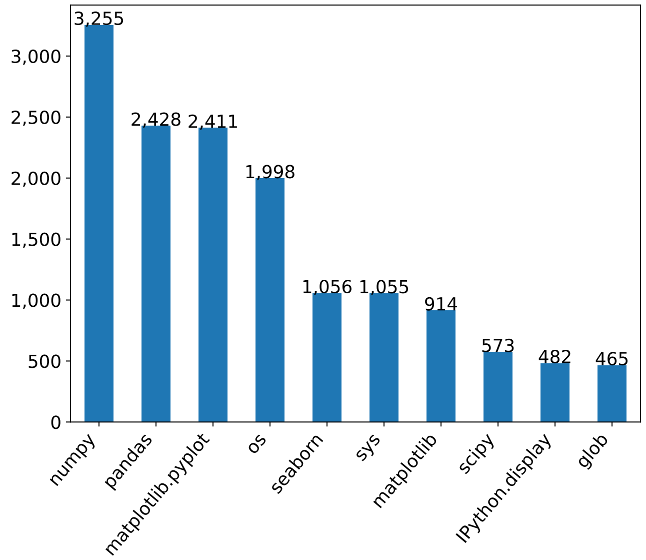

Figure 15. Top Python modules declared in Jupyter Notebooks.

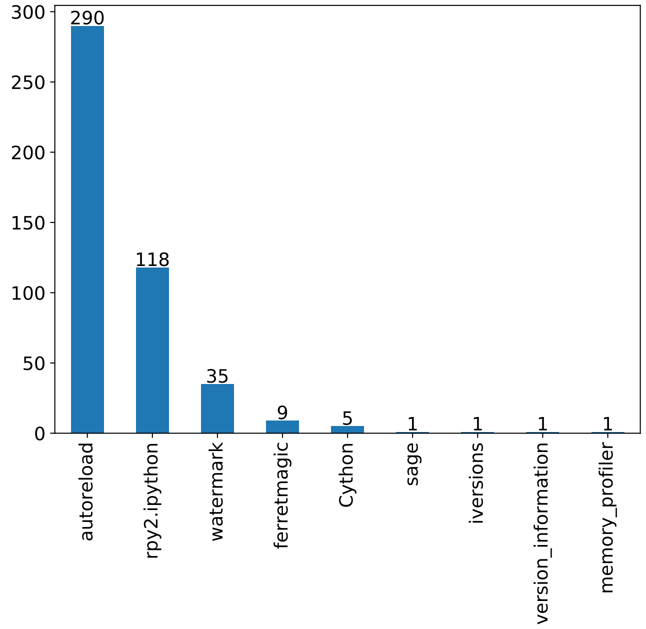

Figure 16. Load extension modules in Jupyter Notebooks

Notebook dependencies

Figure 17 shows the analysis of the declared dependencies of GitHub repositories and notebooks. 4650 (48.31%) of notebooks belong to repositories which have declared dependencies using *setup.py*, *requirements.txt*, or *pipfile*. There are 492 repositories with declared dependencies (Figure 17b). There are 194 repositories with *setup.py* file, 117 repositories with *requirements.txt* file. 180 repositories have both *setup.py* and *requirements.txt* file. Only 10 repositories are with *pipfile* (0.90%). In our study, 3845 (39.95%) of notebooks use *setup.py* file, 2765 (28.73%) notebooks use *requirements.txt* and only 186 (1.93%) notebooks use *pipfile*.

Notebook Reproducibility

In our reproducibility study, we executed 4169 (43.45%) Python notebooks. The dependencies of the notebooks, as mentioned in

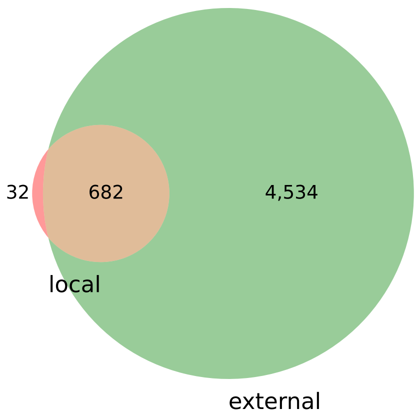

(a) External versus local modules declared in Jupyter notebooks.

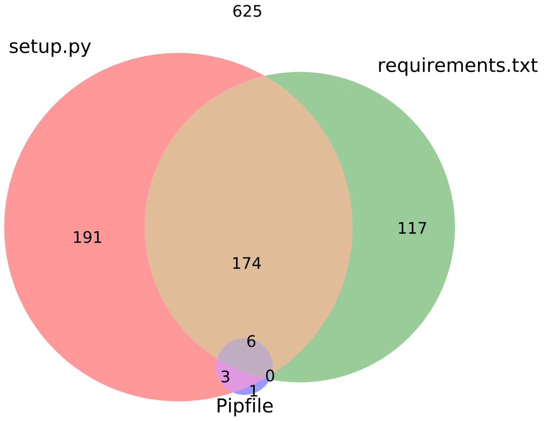

(b) Repositories with dependencies.

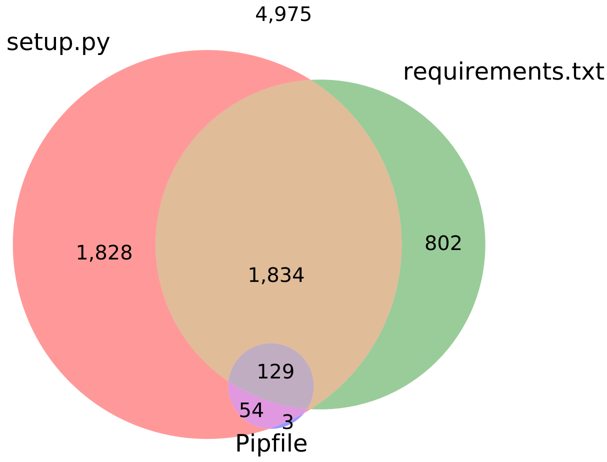

(c) Notebooks with dependencies.

Figure 17. Dependencies of Juypter Notebooks and GitHub repositories. In (a), the notebooks depending on external modules (green) are plotted against notebooks depending on local modules (red) and notebooks that had both (brown). In (b) and (c), GitHub repositories and Jupyter notebooks are shown as to whether they declared their dependencies via any combination of `setup.py` (red), `requirements.txt` (green) or a `pipfile` (pink).

their respective repositories, were installed in conda environments. But, dependencies of 1,485 (35.62%) notebooks failed to install. None of the files were malformed with wrong syntax or conflicting dependencies. We did not find any missing files that required other requirement files which were unavailable or files that needed external tools. Hence, the reason for the failed installed error is unknown. We attempted to execute 2,684 (64.38%) notebooks for the reproducibility study after successfully installing all the requirements. However, many notebooks failed to execute even after installing all the requirements successfully.

Exceptions

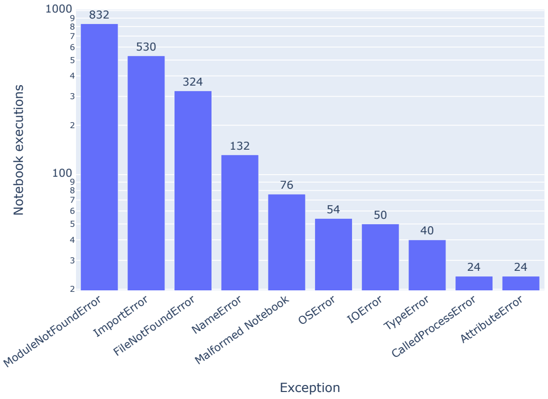

Figure 18. Exceptions occurring in Jupyter Notebooks.

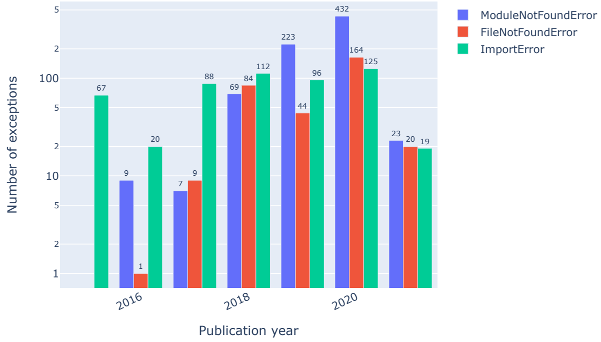

Figure 19. ModuleNotFoundError, ImportError and FileNotFoundError exceptions by year of publication.

2,265 (84.39%) notebooks resulted in exceptions due to several reasons. Figure 18 shows the top ten exceptions that occurred while executing the notebooks. *ModuleNotFoundError*, *ImportError*, and *FileNotFoundError* are the most common reason that resulted in failure of execution in notebooks. 1,362 (32.67%) of the executions failed because of *ModuleNotFoundError* and *ImportError* exceptions. *ModuleNotFoundError* exception occurs when a Python module used by the notebook could not be found. *ImportError* exception occurs when a Python module used by the notebook could not be imported. These two errors occur mainly due to missing dependencies. 132 (3.17%) notebooks have *NameError*, which occurs when a declared variable in the notebook is not defined. 374 (8.97%) notebooks have FileNotFoundError or IOError. These exceptions

occur when absolute paths are used to access data or when the data files are not included in the repository. Figure 19 shows how the top three common exceptions *ModuleNotFoundError*, *ImportError*, and *FileNotFoundError* change with the year the article was published . We see an increase in the *ModuleNotFoundError* through the years. In the years before 2019, the *ImportError* outnumbered *ModuleNotFoundError*.

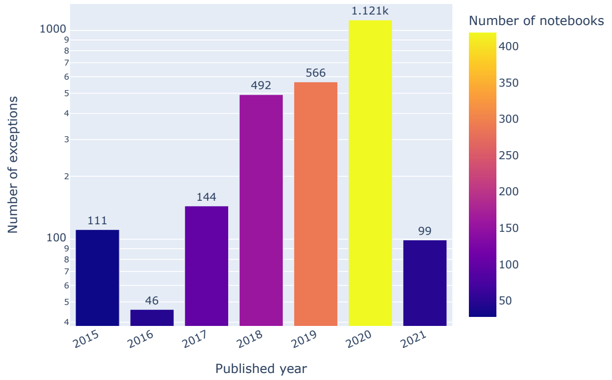

Figure 20. Exceptions by year of publication.

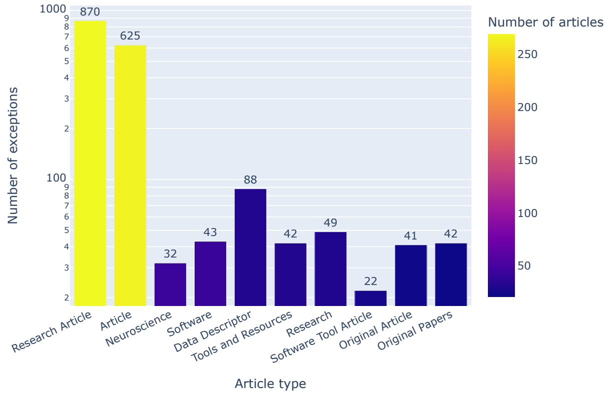

Figure 21. Exceptions by article type.

Figure 20 shows the trend of exceptions by the year of publication normalized by the number of notebooks. In 2020, we observed the highest number of exceptions and notebooks. Figure 21 shows the exceptions by the type of the article. The research articles have the most number of exceptions. Figure 22 shows the exceptions by journal, normalized by the number of notebooks. The journal eLife has the most number of notebooks with the most number of exceptions, followed by PLoS One.

Successful replications

396 (9.50%) of the notebooks in our corpus finished their execution successfully without any errors. However, for 151 notebooks (3.62%), our execution generated results that differed from the original ones, while 245 notebooks (5.88%) produced the same results in our execution as documented for the original notebooks.

Table 1 zooms in on the successfully executed notebooks and

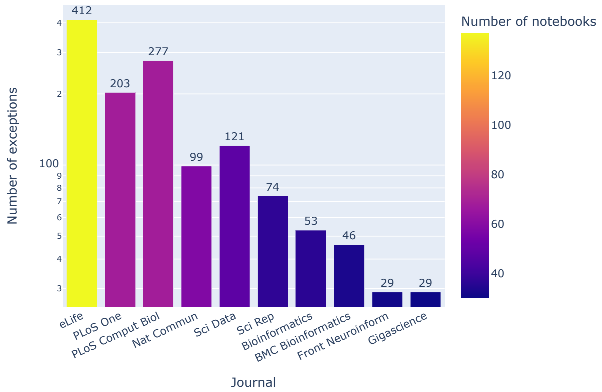

Figure 22. Exceptions by journal.

Table 1. Comparison of notebooks that were successfully executed without errors, grouped by whether their results were `different` from or `identical` to the results documented for the original notebook. For features listed in *italics*, the mean values per notebook are indicated, otherwise totals across all notebooks per group.

Features	Notebooks with `different` results	Notebooks with `identical` results
Number of notebooks	151	245
setup.py	0	98
requirement.txt	0	107
pipfile	0	0
Total cells	17.9	17.1
Code cells	12.3	9.8
Markdown cells	5.6	6.7
Ratio of Markdown vs. code cells	0.46	0.68
Empty cells	0.7	0.7
Differences	5.3	0
Execution time (s)	22.1	16.4
Execution time per code cell (s)	1.80	1.67

compares those that did not yield results the same results as the original ones (`different` group) with those that did (`identical` group). A clear difference between both groups is that many of the notebooks in the `identical` group had their dependencies specified via either setup.py or requirements.txt or both, in contrast to none of the notebooks in the `different` group. Since notebooks with no dependency declarations were run using the default conda dependencies, the fact that they successfully finished means that all dependencies were covered. However, as the version of the dependencies used in the original notebook was not documented, it may have differed from the version that was provided in our respective Conda environment.

Besides versioning of dependencies, there could be a number of other reasons as to why an error-free execution might yield different results. For instance, random functions may be invoked, or code cells in the original might have been executed multiple times or in a different order than in our execution, which ran every code cell just once, from top to bottom. However, we would not expect the invocation of random functions or an inconsistent execution order to correlate so strongly with whether the dependencies had been explicitly declared or not.

In contrast to the dependency declarations, other features in Table 1 show more gradual differences between the two groups, and they largely fit with intuition. For instance, it is understandable that notebooks with more code cells take longer to execute and that

code whose execution per code cell takes longer is somewhat more complex, thus raising the probability of different outcomes. It is also not surprising that, while the total number of cells per notebook is nearly the same in both groups, notebooks in the `identical` group show a higher ratio of Markdown versus code cells, since that ratio is indicative of documentation efforts, and better documentation would be expected to go with better reproducibility.

The average number of differences observed per notebook (or even per code cell) is not easy to interpret on its own, as it includes differences in output cells, cell counter values or in output files, and a difference early in a notebook can lead to further differences later.

Table 2 illustrates how different Python versions performed in terms of successful executions: amongst the top 5 versions for notebooks yielding different results, there were three 2.7 versions, whereas there were three 3.7 versions in the group that yielded identical results, and 3.6.9 and 3.6.5 were represented roughly equally in both groups.

Table 2. Comparison of most frequent Python versions declared for notebooks that were successfully executed without errors, grouped by whether their results were `different` from or `identical` to the results documented for the original notebook. Versions listed in *italics* occur in both top-5 groups, versions listed in **bold** in only one. The `absolute` columns give total number of notebooks per version and group, while the `relative` columns normalize the absolute values as a percentage of the total number of notebooks per group, i.e. 151 for `different` and 245 for `identical`, as per Table 1. In both groups, the top-5 versions account for slightly over half of the notebooks.

	`different`			`identical`		
rank	version	absolute	relative	version	absolute	relative
1	*3.6.9*	28	18.5	**3.7.3**	46	18.8
2	**2.7.6**	17	11.3	*3.6.9*	30	12.2
3	**2.7.10**	13	8.6	**3.7.1**	26	10.6
4	*3.6.5*	12	7.9	**3.7.4**	22	9.0
5	**2.7.9**	11	7.3	*3.6.5*	15	6.1

Other parameters that we considered but did not include in the analysis of the finished notebooks were the number of dependencies (the more there are, the more likely replicability is reduced; see also section Notebook dependencies and in particular Fig. 17), the type of dependencies (e.g. local code or environment, Python package, local or remote file or service, each of which could complicate replication; see also Fig. 15 and 16), the recency (cf. Fig. and 19 and 20) of the notebooks (more recent ones would be expected to be more replicable) or notebook titles (cf. Fig. 12, 13 and 14) containing strings like "tutorial" or "demo" (which might be indicative of expected reuse, thus perhaps triggering more careful documentation) or "untitled" (which is the default title and may thus indicate a lack of attention to documentation and, consequently, a higher likelihood for replication attempts to fail).

Notebook Styling

In addition to the common exceptions in the notebooks, we also checked the notebook code styling errors. Figure 23 shows the most common Python code warning/style errors found in our study.

Table 3 presents the code for the Python code warnings and style errors found in our study. E231 is the most common coding style error, followed by E225 and E265, respectively. There are also some common content errors other than styling errors like F403 and F405 – these are related to variable and module definition errors. The W601 and W606 warnings relate to the use of deprecated and reserved keys.

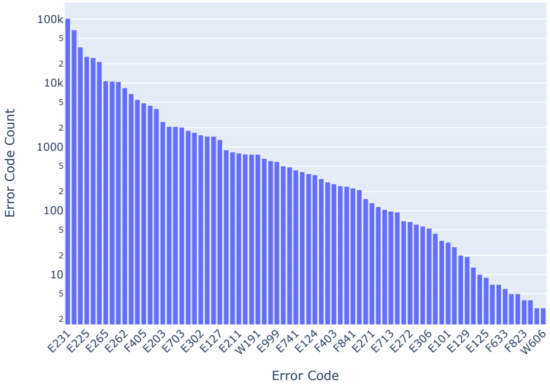

Figure 23. Frequent notebook code style errors as per the Python code style guide.

Table 3. Common Python Notebook Code Warning/Style Error found in our Study

Error code	Description	Count
E231	missing whitespace after commas, semicolons or colons	102218
E225	missing whitespace around operator	25979
E265	block comment should start with '#'	10769
E402	module level import not at top of file	10478
E262	inline comment should start with '#'	8369
E703	statement ends with a semicolon	2023
E127	continuation line over-indented for visual indent	1290
E701	multiple statements on one line	500
E741	do not use variables named 'l', 'O', or 'I'	432
E401	multiple imports on one line	95
E101	indentation contains mixed spaces and tabs	32
F405	*name* may be undefined, or defined from star imports: *module*	4840
F401	*module* imported but unused	3938
F821	undefined name 'X'	2071
F403	'from module import *' used; unable to detect undefined names	263
F841	local variable 'X' is assigned to but never used	225
F404	future import(s) name after other statements	44
F402	import 'X' from line Y shadowed by loop variable	10
F633	use of » is invalid with print function	6
F823	local variable 'X' defined in enclosing scope on line Y referenced before assignment	4
W601	.has_key() is deprecated, use 'in'	7
W606	'async' and 'await' are reserved keywords starting with Python 3.7	3

Discussion

In this study, we have analyzed the *Method reproducibility* – in the sense of Goodman et al. [27] – of Jupyter notebooks written in Python and publicly hosted on GitHub that are mentioned in pub-

lications whose full text was available via PubMed Central by the reference period, i.e. the time when our reproducibility pipeline was run on 24-28 February 2021. We will now discuss the limitations of the study and then its implications, again primarily for *Method reproducibility* of Jupyter notebooks associated with biomedical publications.

Limitations

The present study does not address *Inferential reproducibility* and only briefly touches upon *Results reproducibility*. Furthermore, we made no attempt to re-run computational notebooks that met any of the following exclusion criteria during the reference period: (a) they did not use Jupyter (or its precursor, IPython), (b) they were not written in Python, (c) they were not publicly available on GitHub, (d) they were not mentioned in publications available from PubMed Central, (e) they were not on the base branch of their GitHub repository (which is the only branch we looked at).

Our reproducibility workflow is based on that by [38], with some changes to include GitHub repositories from publications and using the `nbdime` library [68] from Jupyter instead of string matching for finding differences in the notebook outputs. The approach is using conda [69] environments. We did not use any Docker images [70] for the execution environment, even in cases when they were available. This workflow being fully automated, we did not spend any manual effort on fixing any of the errors that came up for an individual notebook – see Woodbridge [71] for a report of an attempt to do so, which also provided the foundation for a prototypical validation tool that makes use of GitLab Actions

For a good number of the reported problems (especially the missing software or data dependencies, as per Fig. 18), it is often straightforward to fix them manually for individual notebooks, yet undertaking manual fixes systematically was not practical at the scale of the thousands of notebooks rerun here. If the original code had specified dependencies without referring to a specific version, our rerun would use the most recent conda-installable version of that library. Finally, in estimating the environmental footprint of this study, we only included the footprint due to running the full pipeline once – we did not include the efforts involved in preparing the pipeline, analyzing the data or writing the manuscript.

Implications

There are several implications of this study. First, on a general level, the low degree of reproducibility that we documented here for Jupyter notebooks associated with biomedical publications goes conform with similarly low levels of reproducibility that were found in earlier domain-generic studies, both for Python [43, 44] and R [36].

Second, considering that the notebooks we explored here were associated with peer-reviewed publications, it is clear that the review processes currently in place at journals within our corpus does not generally pay much attention to the reproducibility of the notebooks. This clearly needs to change, and we need systemic approaches to that rather than just adding this to the list of things the reviewers are expected to attend to. As our study demonstrates, a basic level of reproducibility assessment can well be achieved in a fully automated fashion, so it would probably be beneficial in terms of research quality to include such automated basic checks – for notebooks and other software – into standard review procedures. Ideally, this would be done in a way that works across publishers as well as for a variety of technology stacks and programming languages.

Third, while there is a large variety in the types of errors affecting reproducibility, some of the most common errors concentrate around dependencies (cf. Fig. 15, 16, 17 and 18), so efforts aimed at systemic improvements of dependency handling – e.g. as per [72] – have the potential to increase reproducibility considerably. Here, programming language-specific efforts regarding code dependencies can be combined with efforts targeted at improving the automated handling of data dependencies, which would be beneficial irrespective of the specific programming language.

Fourth, zooming in on Python specifically, wider adoption of existing workflows for code dependency management (such as `requirements.txt`) as well as associated checks during the publishing process would help. Researchers attempting to publish research with associated notebooks should not have to do this all by themselves – research infrastructures as well as publishers and funders can all help facilitate establishing best practice here and engaging communities around them.

Fifth, the few notebooks that actually did reproduce (cf. Successful replications) are not equally distributed. This means that reproducibility could probably be strengthened by enhancing or highlighting the features that correlate with it. For instance, Jupyter notebooks with more emphasis on documentation scored better than others, and there is merit in the idea of making Jupyter notebooks or similar computational notebooks a publication type of their own. This is already the case in some places, as examplified by Constantine et al. [73] or [74] in the Journal of Open Source Software.

Sixth, the ongoing diversification of the Jupyter ecosystem – e.g. in terms of programming languages, deployment frameworks or cloud infrastructure – is increasingly reflected, albeit with delay, in the biomedical literature. In parallel, while GitHub remains hugely popular, alternatives like GitLab, Gitee or Codeberg are growing too. Future assessments of Jupyter reproducibility will thus need to take this increasing complexity into account, and ideally present some systematic approach to it.

Seventh, the delays that come with current publishing practices also mean that Jupyter notebooks associated with freshly published papers are using software versions near or even beyond their respective support window (which is 42 months in much of the Python ecosystem[23]).

For instance, the oldest Python version still officially supported in 2021 was 3.6 (which was itself retired by the end of 2021, when 3.10 was released[24]), yet as shown in Figure 9, over a thousand Python notebooks in our corpus whose last commit was in 2021 still featured earlier Python versions, mainly 2.7 (outphased in 2020) but also 3.4 (2019), 3.5 (2020) and some for which the version could not be determined. This contributes to reproducibility issues. A similar issue exists with the versions of the libraries called from any given notebook, though the effects might differ as a function of whether they have been invoked with or without the version being specified. If the version had been specified, its official end of life might go back even further. If the version was not specified, the newest available version would be invoked, which may not be compatible with the way the library had been used in the original notebook. Similar issues can arise with the versioning of APIs, datasets, ontologies or other standards used in the notebook, all of which can contribute to reduced reproducibility. To some extent, these version delay issues can be shortened by preprints: since they are (essentially by definition, but not always in practice) published before the final version of the associated manuscript, and hence their delays should be shorter, with lower reductions in reproducibility, though we did not investigate that in detail.

Eight, the variety and scale of issues encountered in the notebooks analyzed here provides ample opportunities for use in educational contexts – including instructed, self-guided or group learning – since fixing real-life bugs can be more motivating than working primarily with textbook examples. To do this effectively would require some mapping of the strengths and weaknesses of

23 Cf. https://numpy.org/neps/nep-0029-deprecation_policy.html
24 See https://endoflife.date/python for release schedule

the notebooks to learning objectives, which may range from understanding programming paradigms, software engineering principles or data integration workflows to developing an appreciation for documentation and other aspects of good scientific practice. Given the continuously expanding breadth of publications that use Jupyter notebooks, it is also steadily becoming easier to find publications where they have been used in research meeting specific criteria. These could be a particular topic – e.g. natural products research [75] or invasion biology [76] – or workflows involving a particular experimental methodology like single-cell RNA sequencing [77] or other software tools like ImageJ [78].

Conclusions

On the basis of re-running 4169 Jupyter notebooks associated with 1419 publications whose full text is available via Pubmed Central, we conclude that such notebooks are becoming more and more popular for sharing code associated with biomedical publications, that the range of programming languages or journals they cover is continuously expanding and that their reproducibility is low but improving, consistent with earlier studies on Jupyter notebooks shared in other contexts. The main issues are related to dependencies – both code and data – which means that reproducibility could likely be improved considerably if the code – and dependencies in particular – were better documented. Further improvements could be expected if some basic and automated reproducibility checks of the kind performed here were to be systematically included in the peer review process or if computational notebooks – Jupyter or otherwise – were combined with additional approaches that address reproducibility from other angles, e.g. registered reports.

Data availability

All the data generated during this study is available at https://doi.org/10.5281/zenodo.6802158 [79]. The code used is available at https://github.com/fusion-jena/computational-reproducibility-pmc. The code contains notebooks used for analysis of the results.

Ethical Approval (optional)

No facet of the research reported here triggered a requirement for ethical review. While our data contains personally identifiable information, it was taken directly from PMC. We did, however, consider the ethical implications of automated reproducibility studies of the kind presented here, which led us to (a) highlight systemic aspects, (b) not zoom in on individual stakeholders other than journals and (c) include environmental footprint information.

Consent for publication

Not applicable.

Competing Interests

The authors declare there are no competing interests.

Funding

Work by S.S. was supported by the Carl Zeiss Foundation for the project "A Virtual Werkstatt for Digitization in the Sciences (K3)" [80] within the scope of the program line "Breakthroughs: Exploring Intelligent Systems for Digitization - explore the basics, use applications". Work by D.M. was supported by the Alfred P. Sloan Foundation under grant number G-2021-17106 [81]. The computational experiments were performed on resources of Friedrich Schiller University Jena supported in part by DFG grants INST 275/334-1 FUGG and INST 275/363-1 FUGG.

Author's Contributions

S.S conceived and designed the experiments, performed the experiments, analyzed the data, prepared figures and/or tables, authored or reviewed drafts of the paper, and approved the final draft.

D.M. conceived and designed the experiments, authored or reviewed drafts of the paper, analyzed the data, prepared figures and/or tables, and approved the final draft.

Acknowledgements

We would like to thank the providers of infrastructure, data and code that we used in this study. These include the PubMed Central repository hosted by the National Center for Biotechnology Information in the United States and the Ara Cluster at the University of Jena as well as the Python, Jupyter and Conda communities and their respective dependencies. We acknowledge the Open Research Doathon on the occasion of Open Data Day 2017, where the first attempts at systematic reproduction of PMC-indexed Jupyter notebooks were made [71]. Special thanks go to JupyterCon, which made the two of us aware of each other's work and provided the nucleus for our collaboration.

Authors' information (optional)

S.S. is a postdoctoral researcher at the Friedrich Schiller University Jena, Germany. Her work focus on e-Science, Semantic Web and Machine Learning, specifically on improving reproducibile research using provenance, linked data, etc.

D.M. is a biophysicist working on integrating open research and education workflows with the web, for all stages of the research cycle. His research activities span across the spatial and temporal scales of life and concentrate on the data aspects of research, particularly in the life sciences and adjacent fields.

References

1. Siebert S, Machesky LM, Insall RH. Point of view: Overflow in science and its implications for trust. Elife 2015;4:e10825.
2. Contera S. Communication is central to the mission of science. Nature Reviews Materials 2021;6(5):377–378.
3. Gray S, Shwom R, Jordan R. Understanding factors that influence stakeholder trust of natural resource science and institutions. Environmental management 2012;49(3):663–674.
4. Kroeger CM, Garza C, Lynch CJ, Myers E, Rowe S, Schneeman BO, et al. Scientific rigor and credibility in the nutrition research landscape. The American journal of clinical nutrition 2018 March;107(3):484—494. https://europepmc.org/articles/PMC6248649.
5. Jamieson KH, McNutt M, Kiermer V, Sever R. Signaling the trustworthiness of science. Proceedings of the National Academy of Sciences 2019;116(39):19231–19236. https://www.pnas.org/content/116/39/19231.
6. Hsieh T, Vaickus MH, Remick DG. Enhancing scientific foundations to ensure reproducibility: a new paradigm. The American journal of pathology 2018;188(1):6–10.
7. Peng R. The reproducibility crisis in science: A statistical counterattack. Significance 2015;12(3):30–32.
8. Samuel S, König-Ries B. Understanding experiments and re-

search practices for reproducibility: an exploratory study. PeerJ 2021 Apr;9:e11140. https://doi.org/10.7717/peerj.11140.
9. The Economist, Trouble at the lab; 2013. https://www.economist.com/briefing/2013/10/18/trouble-at-the-lab.
10. Simmons JP, Nelson LD, Simonsohn U. False-Positive Psychology: Undisclosed Flexibility in Data Collection and Analysis Allows Presenting Anything as Significant. Psychological Science 2011 oct 17;22(11):1359–1366.
11. Hussain W, Moens N, Veraitch FS, Hernandez D, Mason C, Lye GJ. Reproducible Culture and Differentiation of Mouse Embryonic Stem Cells Using an Automated Microwell Platform. Biochemical engineering journal 2013;77(100):246–257.
12. Bairoch AM. The Cellosaurus, a Cell-Line Knowledge Resource. Journal of Biomolecular Techniques 2018;29(2):25–38.
13. Kelly CD. Rate and success of study replication in ecology and evolution. PeerJ 2019 sep 10;7:e7654.
14. Ledermann F, Gartner G. Towards Conducting Reproducible Distributed Experiments in the Geosciences. AGILE: GIScience Series 2021 jun 4;2:1–7.
15. Coiera EW, Ammenwerth E, Georgiou A, Magrabi F. Does health informatics have a replication crisis? Journal of the American Medical Informatics Association 2018 aug 1;25(8):963–968.
16. Hinsen K. Verifiability in computer-aided research: the role of digital scientific notations at the human-computer interface. PeerJ Computer Science 2018;4:e158.
17. Hutson M. Artificial intelligence faces reproducibility crisis. Science 2018;359(6377):725–726. https://science.sciencemag.org/content/359/6377/725.
18. Shepperd M, Ajienka N, Counsell S. The role and value of replication in empirical software engineering results. Information and Software Technology 2018 7;99:120–132.
19. Crick T, Hall BA, Ishtiaq S. Reproducibility in Research: Systems, Infrastructure, Culture. Journal of open research software 2017 nov 9;5(1):32.
20. Baker M. 1,500 scientists lift the lid on reproducibility. Nature 2016 may 25;533(7604):452–454.
21. Hunter P. The reproducibility "crisis". EMBO reports 2017;18(9):1493–1496. https://www.embopress.org/doi/abs/10.15252/embr.201744876.
22. Fanelli D. Opinion: Is Science Really Facing a Reproducibility Crisis, and Do We Need It To? Proceedings of the National Academy of Sciences of the United States of America 2018;115(11):2628–2631.
23. Guttinger S. The limits of replicability. European journal for philosophy of science 2020 jan 15;10(2).
24. Näpflin K, O'Connor EA, Becks L, Bensch S, Ellis VA, Hafer-Hahmann N, et al., Genomics of host-pathogen interactions: challenges and opportunities across ecological and spatiotemporal scales; 2019.
25. Meng XL. Reproducibility, Replicability, and Reliability. Harvard Data Science Review 2020 oct 29;2(4). Https://hdsr.mitpress.mit.edu/pub/hn51kn68.
26. Plesser HE. Reproducibility vs. Replicability: A Brief History of a Confused Terminology. Frontiers in Neuroinformatics 2017 jan 1;11:76.
27. Goodman SN, Fanelli D, Ioannidis JPA. What does research reproducibility mean? Science Translational Medicine 2016 jun 1;8(341):341ps12.
28. Burlingame EA, Eng J, Thibault G, Chin K, Gray JW, Chang YH. Toward reproducible, scalable, and robust data analysis across multiplex tissue imaging platforms. Cell Reports Methods 2021 8;1(4):100053.
29. Russell PH, Johnson RL, Ananthan S, Harnke B, Carlson NE. A large-scale analysis of bioinformatics code on GitHub. PLOS ONE 2018;13(10):e0205898.
30. Sandve GK, Nekrutenko A, Taylor J, Hovig E. Ten Simple Rules for Reproducible Computational Research. PLOS Computational Biology 2013 10;9(10):1–4. https://doi.org/10.1371/journal.pcbi.1003285.
31. Gil Y, David CH, Demir I, Essawy BT, Fulweiler RW, Goodall JL, et al. Toward the Geoscience Paper of the Future: Best practices for documenting and sharing research from data to software to provenance. Earth and Space Science 2016;3(10):388–415. https://agupubs.onlinelibrary.wiley.com/doi/abs/10.1002/2015EA000136.
32. Willcox A, ReSearchOps: a principled framework and guide to computational reproducibility. Open Science Framework; 2021.
33. Grüning B, Chilton J, Köster J, Dale R, Soranzo N, van den Beek M, et al. Practical Computational Reproducibility in the Life Sciences. Cell systems 2018 jun 1;6(6):631–635.
34. Brito JJ, Li J, Moore JH, Greene CS, Nogoy NA, Garmire LX, et al. Recommendations to enhance rigor and reproducibility in biomedical research. GigaScience 2020 06;9(6). https://doi.org/10.1093/gigascience/giaa056, giaa056.
35. Nüst D, Sochat VV, Marwick B, Eglen SJ, Head T, Hirst T, et al. Ten simple rules for writing Dockerfiles for reproducible data science. PLOS Computational Biology 2020 nov 10;16(11):e1008316.
36. Trisovic A, Lau MK, Pasquier T, Crosas M. A large-scale study on research code quality and execution. Scientific Data 2022 feb 21;9(1):60.
37. Rule A, Birmingham A, Zuniga C, Altintas I, Huang S, Knight R, et al. Ten simple rules for writing and sharing computational analyses in Jupyter Notebooks. Plos Computational Biology 2019;15(7):e1007007–e1007007.
38. Pimentel JaF, Murta L, Braganholo V, Freire J. A Large-scale Study About Quality and Reproducibility of Jupyter Notebooks. In: Proceedings of the 16th International Conference on Mining Software Repositories MSR '19, Piscataway, NJ, USA: IEEE Press; 2019. p. 507–517.
39. Wang J, Kuo Ty, Li L, Zeller A. Restoring Reproducibility of Jupyter Notebooks. In: 2020 IEEE/ACM 42nd International Conference on Software Engineering: Companion Proceedings (ICSE-Companion); 2020. p. 288–289.
40. Willis A, Charlton P, Hirst T. Developing students' written communication skills with Jupyter notebooks. In: Proceedings of the 51st ACM Technical Symposium on Computer Science Education; 2020. p. 1089–1095.
41. Wang J, Li L, Zeller A. Better code, better sharing: on the need of analyzing jupyter notebooks. In: Proceedings of the ACM/IEEE 42nd International Conference on Software Engineering: New Ideas and Emerging Results; 2020. p. 53–56.
42. Halchenko YO, Meyer K, Poldrack B, Solanky DS, Wagner AS, Gors J, et al. DataLad: distributed system for joint management of code, data, and their relationship. Journal of Open Source Software 2021;6(63):3262. https://doi.org/10.21105/joss.03262.
43. Rule A, Tabard A, Hollan JD. Exploration and Explanation in Computational Notebooks. In: Proceedings of the 2018 CHI Conference on Human Factors in Computing Systems CHI '18, New York, NY, USA: ACM; 2018. p. 32:1–32:12.
44. Pimentel JF, Murta L, Braganholo V, Freire J. Understanding and improving the quality and reproducibility of Jupyter notebooks. Empir Softw Eng 2021;26(4):65. https://doi.org/10.1007/s10664-021-09961-9.
45. Roberts RJ. PubMed Central: The GenBank of the published literature. Proceedings of the National Academy of Sciences 2001;98(2):381–382. https://www.pnas.org/content/98/2/381.
46. Sayers E. A General Introduction to the E-utilities. Entrez Programming Utilities Help [Internet] Bethesda (MD): National Center for Biotechnology Information (US) 2010;.
47. Kluyver T, Ragan-Kelley B, Pérez F, Granger B, Bussonnier M, Frederic J, et al. Jupyter Notebooks-a publishing format for reproducible computational workflows. In: ELPUB; 2016. p. 87–90.

48. Granger BE, Perez F. Jupyter: Thinking and Storytelling With Code and Data. Computing in Science and Engineering 2021 mar 26;23(2):7–14.
49. Randles BM, Pasquetto IV, Golshan MS, Borgman CL. Using the Jupyter notebook as a tool for open science: An empirical study. In: 2017 ACM/IEEE Joint Conference on Digital Libraries (JCDL) IEEE; 2017. p. 1–2.
50. Wofford MF, Boscoe BM, Borgman CL, Pasquetto IV, Golshan MS. Jupyter notebooks as discovery mechanisms for open science: Citation practices in the astronomy community. Computing in Science & Engineering 2019;22(1):5–15.
51. Schröder M, Krüger F, Spors S. Reproducible Research is more than Publishing Research Artefacts: A Systematic Analysis of Jupyter Notebooks from Research Articles. CoRR 2019;abs/1905.00092. http://arxiv.org/abs/1905.00092.
52. Chattopadhyay S, Prasad I, Henley AZ, Sarma A, Barik T. What's Wrong with Computational Notebooks? Pain Points, Needs, and Design Opportunities. In: Proceedings of the 2020 CHI Conference on Human Factors in Computing Systems; 2020. p. 1–12.
53. Chirigati F, Shasha D, Freire J. ReproZip: Using Provenance to Support Computational Reproducibility. In: Presented as part of the 5th USENIX Workshop on the Theory and Practice of Provenance Lombard, IL: USENIX; 2013. https://www.usenix.org/conference/tapp13/reprozip-using-provenance-support-computational-reproducibility.
54. Boettiger C. An Introduction to Docker for Reproducible Research. SIGOPS Oper Syst Rev 2015 Jan;49(1):71–79. http://doi.acm.org/10.1145/2723872.2723882.
55. Samuel S, König-Ries B. ProvBook: Provenance-based Semantic Enrichment of Interactive Notebooks for Reproducibility. In: van Erp M, Atre M, López V, Srinivas K, Fortuna C, editors. Proceedings of the ISWC 2018 Posters & Demonstrations, Industry and Blue Sky Ideas Tracks co-located with 17th International Semantic Web Conference (ISWC 2018), Monterey, USA, October 8th - to - 12th, 2018, vol. 2180 of CEUR Workshop Proceedings CEUR-WS.org; 2018. http://ceur-ws.org/Vol-2180/paper-57.pdf.
56. Project Jupyter, Bussonnier M, Forde J, Freeman J, Granger BE, Head T, et al. Binder 2.0 - Reproducible, interactive, sharable environments for science at scale. In: Proceedings of the 17th Python in Science Conference; 2018. p. 113 – 120.
57. Baker AM, Cereser B, Melton S, Fletcher AG, Rodriguez-Justo M, Tadrous PJ, et al. Quantification of Crypt and Stem Cell Evolution in the Normal and Neoplastic Human Colon. Cell Reports 2019 may 1;27(8):2524.
58. Meyerowitz-Katz G, Besançon L, Flahault A, Wimmer R. Impact of mobility reduction on COVID-19 mortality: absence of evidence might be due to methodological issues. Scientific Reports 2021 dec 7;11(1).
59. Waagmeester A, Stupp G, Burgstaller-Muehlbacher S, Good BM, Griffith M, Griffith O, et al. Wikidata as a knowledge graph for the life sciences. eLife 2020 mar 17;9.
60. Rutz A, Sorokina M, Galgonek J, Mietchen D, Willighagen E, Gaudry A, et al. The LOTUS initiative for open knowledge management in natural products research. eLife 2022 may 26;11.
61. Nielsen FÅ, Mietchen D, Willighagen E. Scholia, Scientometrics and Wikidata. In: The Semantic Web: ESWC 2017 Satellite Events; 2017. p. 237–259.
62. Lannelongue L, Grealey J, Inouye M. Green Algorithms: Quantifying the Carbon Footprint of Computation. Advanced Science 2021;n/a(n/a):2100707. https://onlinelibrary.wiley.com/doi/abs/10.1002/advs.202100707.
63. Taddeo M, Tsamados A, Cowls J, Floridi L. Artificial intelligence and the climate emergency: Opportunities, challenges, and recommendations. One Earth 2021;4(6):776–779.
64. Schwartz R, Dodge J, Smith NA, Etzioni O. Green AI. Communications of the ACM 2020 nov 17;63(12):54–63.
65. Lannelongue L, Grealey J, Bateman A, Inouye M. Ten simple rules to make your computing more environmentally sustainable. PLOS Computational Biology 2021 sep 20;17(9):e1009324.
66. Cock PJA, Antao T, Chang JT, Chapman BA, Cox CJ, Dalke A, et al. Biopython: freely available Python tools for computational molecular biology and bioinformatics. Bioinformatics 2009 03;25(11):1422–1423. https://doi.org/10.1093/bioinformatics/btp163.
67. Samuel S, König-Ries B. ReproduceMeGit: A Visualization Tool for Analyzing Reproducibility of Jupyter Notebooks. In: Glavic B, Braganholo V, Koop D, editors. Provenance and Annotation of Data and Processes Cham: Springer International Publishing; 2021. p. 201–206.
68. Project Jupyter, nbdime: Jupyter Notebook Diff and Merge tools; 2021. Accessed 18 May 2021. https://github.com/jupyter/nbdime.
69. Conda community, Conda; 2017. https://conda.io/.
70. Docker, Docker; 2013. https://www.docker.com.
71. Woodbridge M, Jupyter Notebooks and reproducible data science; 2017. https://markwoodbridge.com/2017/03/05/jupyter-reproducible-science.html.
72. Zhu C, Saha RK, Prasad MR, Khurshid S. Restoring the Executability of Jupyter Notebooks by Automatic Upgrade of Deprecated APIs. In: 2021 36th IEEE/ACM International Conference on Automated Software Engineering (ASE); 2021. p. 240–252.
73. Constantine P, Howard R, Glaws A, Grey Z, Diaz P, Fletcher L. Python Active-subspaces Utility Library. Journal of Open Source Software 2016 sep 29;1(5):79.
74. Garg A, Smith-Unna RD, Murray-Rust P. pygetpapers: a Python library for automated retrieval of scientific literature. Journal of Open Source Software 2022 jul 7;7(75):4451.
75. Mayr F, Möller G, Garscha U, Fischer J, Castaño PR, Inderbinen SG, et al. Finding New Molecular Targets of Familiar Natural Products Using In Silico Target Prediction. International Journal of Molecular Sciences 2020 sep 26;21(19).
76. Bors EK, Herrera S, Morris JA, Shank TM. Population genomics of rapidly invading lionfish in the Caribbean reveals signals of range expansion in the absence of spatial population structure. Ecology and evolution 2019 feb 10;9(6):3306–3320.
77. Vargo AHS, Gilbert AC. A rank-based marker selection method for high throughput scRNA-seq data. BMC Bioinformatics 2020 oct 23;21(1):477.
78. Bryson AE, Brown MW, Mullins J, Dong W, Bahmani K, Bornowski N, et al. Composite modeling of leaf shape along shoots discriminates Vitis species better than individual leaves. Applications in plant sciences 2020 dec 3;8(12):e11404.
79. Samuel S, Mietchen D, Dataset of a Study of Computational reproducibility of Jupyter notebooks from biomedical publications; 2022. https://zenodo.org/record/6802158.
80. Samuel S, Shadaydeh M, Böcker S, Brügmann B, Bucher SF, Deckert V, et al. A virtual "Werkstatt" for digitization in the sciences. Research Ideas and Outcomes 2020 may 11;6.
81. Rasberry L, Mietchen D. Scholia for Software. Research Ideas and Outcomes 2022 sep 15;8.
