# Supplementary material for: Computational reproducibility of Jupyter notebooks from biomedical publications
Source: Gigascience. 2024 Jan 11;13:giad113. doi: 10.1093/gigascience/giad113 (PMC10783158; doi:10.1093/gigascience/giad113)
Supplement: giad113_Response_to_Reviewer_Comments_Original_Submission [file giad113_response_to_reviewer_comments_original_submission.pdf]

Author's Response To Reviewer Comments

Our responses are provided in the file Rebuttal-GIGA-D-22-00259_Jupyter_reproducibility-2023-08-09.pdf
.
