# Supplementary material for: Computational reproducibility of Jupyter notebooks from biomedical publications
Source: Gigascience. 2024 Jan 11;13:giad113. doi: 10.1093/gigascience/giad113 (PMC10783158; doi:10.1093/gigascience/giad113)
Supplement: giad113_Response_to_Reviewer_Comments_Revision_1 [file giad113_response_to_reviewer_comments_revision_1.pdf]

Author's Response To Reviewer Comments

This version addresses all the editorial issues listed in the Decision Letter.
