# Supplementary material for: Computational reproducibility of Jupyter notebooks from biomedical publications
Source: Gigascience. 2024 Jan 11;13:giad113. doi: 10.1093/gigascience/giad113 (PMC10783158; doi:10.1093/gigascience/giad113)
Supplement: giad113_Reviewer_1_Report_Original_Submission — Timothy M. Errington, Ph.D -- 11/7/2022 Reviewed [file giad113_reviewer_1_report_original_submission.pdf]

Reviewer Report

Title: Computational reproducibility of Jupyter notebooks from biomedical publications

Version: Original Submission **Date:** 11/7/2022

Reviewer name: Timothy M. Errington, Ph.D.

Reviewer Comments to Author:

This manuscript reports a large-scale effort to assess the reproducibility of publication associated Jupyter notebooks. It describes in detail the diversity of implementations and associated inconsistences when trying to rerun the notebooks. The authors also suggest several suggestions to improve reproducibility. Below are suggestions for the authors to consider when revising the manuscript:
1) Last sentence of first paragraph in Introduction - the authors refer to reproductions (re-analyze of the same code and data), but then shift to the word replication study. Same with the first sentence in the second paragraph and the paragraph on Jupyter and reproducibility. I suggest the authors keep the nomenclature aligned in this manuscript and making it reproduction in all cases where they are referencing studies similar to what this manuscript is about despite the flexibility of the term being used in cited papers, as the authors highlight in the third paragraph of the Introduction.
2) The section 'Wikidata' in the introduction needs to be integrated more - it lacks context and seems disjoined from the rest of the Introduction. It might make sense to remove it from the Introduction and place it where context is relevant similar to ORCID.
3) Instead of listing the numbers in the text of what the total articles were that were searched and included/excluded, I suggest the authors use the PRISMA flow diagram (https://prisma-statement.org/prismastatement/flowdiagram.aspx).
4) I'm surprised the authors didn't break out any of the characteristics presented in figures 2-5 (in particular) by field? That is more informative instead of journal, especially when considering that many of these journals cover multiple disciplines. Is there a reason for not showing this? If not, I suggest adding.
5) The authors should consider including eLife's executable research articles in the context of the discussion (https://elifesciences.org/for-the-press/eb096af1/elife-launches-executable-research-articles-for-publishing-computationally-reproducible-results & https://elifesciences.org/labs/ad58f08d/introducing-elife-s-first-computationally-reproducible-article).
6) While the authors include the limitation of not including any manual checks, they did list several assumptions in their approach (e.g., only searching the base branch). If possible, it would strengthen the argument if they checked a random sample of articles to test these assumptions and/or reach out to authors to share the findings and understand what it would take to achieve consistency. It would also provide insight into the effort to either fix these errors, to improve the approach the authors deployed to assess reproducibility at scale, and to highlight improvements in the research ecosystem (e.g., specific aspects to focus on in trainings)?

Methods

Are the methods appropriate to the aims of the study, are they well described, and are necessary controls included? Choose an item.

Conclusions

Are the conclusions adequately supported by the data shown? Choose an item.

Reporting Standards

Does the manuscript adhere to the journal's guidelines on minimum standards of reporting? Choose an item.

Choose an item.

Statistics

Are you able to assess all statistics in the manuscript, including the appropriateness of statistical tests used? Choose an item.

Quality of Written English

Please indicate the quality of language in the manuscript: Choose an item.

Declaration of Competing Interests

Please complete a declaration of competing interests, considering the following questions:

- Have you in the past five years received reimbursements, fees, funding, or salary from an organisation that may in any way gain or lose financially from the publication of this manuscript, either now or in the future?
- Do you hold any stocks or shares in an organisation that may in any way gain or lose financially from the publication of this manuscript, either now or in the future?
- Do you hold or are you currently applying for any patents relating to the content of the manuscript?
- Have you received reimbursements, fees, funding, or salary from an organization that holds or has applied for patents relating to the content of the manuscript?
- Do you have any other financial competing interests?
- Do you have any non-financial competing interests in relation to this paper?

If you can answer no to all of the above, write 'I declare that I have no competing interests' below. If your reply is yes to any, please give details below.

I am an employee of the nonprofit Center for Open Science (COS) that has a mission to increase openness, integrity, and reproducibility of research.

I agree to the open peer review policy of the journal. I understand that my name will be included on my report to the authors and, if the manuscript is accepted for publication, my named report including any attachments I upload will be posted on the website along with the authors' responses. I agree for my report to be made available under an Open Access Creative Commons CC-BY license (http://creativecommons.org/licenses/by/4.0/). I understand that any comments which I do not wish to be included in my named report can be included as confidential comments to the editors, which will not be published.

Choose an item.

To further support our reviewers, we have joined with Publons, where you can gain additional credit to further highlight your hard work (see: https://publons.com/journal/530/gigascience). On publication of this paper, your review will be automatically added to Publons, you can then choose whether or not to claim your Publons credit. I understand this statement.

Yes Choose an item.
