# Supplementary material for: Computational reproducibility of Jupyter notebooks from biomedical publications
Source: Gigascience. 2024 Jan 11;13:giad113. doi: 10.1093/gigascience/giad113 (PMC10783158; doi:10.1093/gigascience/giad113)
Supplement: giad113_Reviewer_2_Report_Original_Submission — Ryan Dale -- 11/9/2022 Reviewed [file giad113_reviewer_2_report_original_submission.pdf]

Reviewer Report

Title: Computational reproducibility of Jupyter notebooks from biomedical publications

Version: Original Submission **Date:** 11/9/2022

Reviewer name: Ryan Dale

Reviewer Comments to Author:

The authors identify almost 10k Jupyter notebooks, evaluate them for reproducibility in a high-throughput manner, and report on various metrics from doing so. They take advantage of code provided by previous authors to implement this.

In general, this is a very interesting dataset. What is presented in the current version at times feels superfluous (e.g., distributions of titles and style errors) but at the same time stops short of showing *relationships* among the metrics. For example, this data set can be used to test the hypothesis that title length correlates (or not) with reproducibility. Logistic regression coefficients between reproducibility status and various metrics can be used to indicate which of the metrics are most predictive of reproducibility, and can perhaps be used to e.g. infer decay rates ("every passing year decreases probability of reproducing by X%"). I feel like there is a lot here remaining to be discovered and to add value to the manuscript!

Minor text comments:

* Abstract: sentence one seems to be missing a word : "Jupyter notebooks allow to bundle executable code …", change to something like "Jupyter notebooks allow developers to bundle executable code.."
* "software-affine" seems like an odd word choice to me
* iPython -> IPython
* "it was followed by plos one..." bottom left pg 7 missing sentence?
* Stylistically, I would prefer making conclusions/interpretations and then citing the figure, rather than the "fig N shows" style, to help tie the story together

Suggestions/questions/clarifications:

* Data on 2021 notebooks should be removed from figures; since this analysis was run in February 2021, the data for 2021 is incomplete and distracts from complete years
* The first paragraph of results mentions that 49 of the Github repo links were not accessible, could you rerun just this portion of the pipeline to see how access to the repos change over time? (I.e., might expect that even more repositories from this pool have been moved/are no longer accessible the further out you go from publication date)
* Programming Languages section: It's mentioned that notebooks in the "Unknown" section may be early notebooks for which Python was hardcoded. Could you dig further into this to define 1) when was python no longer hardcoded/PL specifications became common practice? 2) How many of these "unknown" language notebooks were published/last updated before this shift? This information could give more context on how many notebooks truly have "Unknown" language or if they're being evaluated by standards of PL specification that didn't exist at the time of their writing
* Given that missing dependencies were such a big issue, can you look for "import" statements to install

implied dependencies? The AST is already being parsed, so hopefully it would be a straightforward addition. That might dramatically improve the reproducibility rate, and would not otherwise require manual intervention
* It would be interesting to see a flowchart showing the "attrition rate" as the authors went through the pipeline. Or maybe an upset plot showing shared properties. E.g, most "Untitled.ipynb" also have import errors (or something along those lines). Relationships between metrics are not addressed at all.
* On average, a repo had 9 notebooks? (top of pg 7). That's surprising. If one notebook in a repo reproduced, did all of them? Perhaps a scatter plot of number of notebooks vs percent reproduced, with points colored by number of occurrences of that number/percent pair.
* The difference is unclear between "repositories", "repositories with notebooks", and "just notebooks". Does the latter imply no associated repository? pg 7 middle right suggests this is the case but it would be helpful to clarify
* Unclear on the rationale or relevance of ORCID here. Is it just that they are both new-ish and so maybe you're using ORCID as some sort of control or expectation-setter for uptake rate of notebooks? If so, then other "things that happened around the same time" should be used for more context, if you want to interpret the ORCID results.
* Cell count and composition, title length, and human language to me seem irrelevant to reproducibility and could be omitted. But this hypothesis could be tested with the existing dataset!
* The "untitled" notebooks I think I would associate with non-reproducibility; but this can/should be tested in this dataset.
* Were conda environment specifications (as env.yml files) looked for when finding dependencies? That could be another way of solving dependency issues.
* Recency was deliberately excluded as a metric, but I would like to see that reported. It would be interesting to see what the "reproducibility decay rate" is.
* For research computing, while I am personally a strong proponent of style, I don't think this necessarily correlates with reproducibility. But again you have the data to demonstrate whether it correlates or not.
* Exclusion criteria was "not written in python" yet many other languages are shown in Fig 7. So these are notebooks that are in Python, but also have other cell types? That could be clarified somewhere.
Figures & tables
* Unclear what the colors mean in Fig 1
* Figure 3 and 5 could be made larger by moving the legend, they're both a bit small
* Fig 4 would be better as scatterplot and maybe log scale
* Make year labeling consistent (e.g., 6 and 8)
* Figure 9 - can the version release dates be added on the version legend? Could help give perspective on when these different versions began to be adopted. Also hard to differentiate color for 3.4 vs 3.9 (though it appears we can assume it is ordered by legend)
* Fig 10 not discussed, but possibly more informative than Fig 9 (and implies that the notebooks published later are from older code)
* Interpretation of Fig 13 is unclear. Are these bad names? E.g., "Index.ipynb" is one of the names, but the repo for this very paper has an Index.ipynb.
* Fig 20 should be scatter
* Fig 22 should be percentage failed

* Can remove Figure 23 since all information represented by this figure is captured in Table 3. If you want a more clear ranking of error code representation, you could add an additional % of errors column
* Fix formatting overlap between Tables 2 & 3
* For Table 2, I'd change the column labels from absolute and relative to something like notebook count & notebook percentage. Absolute and relative give a different connotation (ex: absolute made me think on first glance this was a pinned version of Python described for the notebook environment)

Methods

Are the methods appropriate to the aims of the study, are they well described, and are necessary controls included? Choose an item.

Conclusions

Are the conclusions adequately supported by the data shown? Choose an item.

Reporting Standards

Does the manuscript adhere to the journal's guidelines on minimum standards of reporting? Choose an item.

Choose an item.

Statistics

Are you able to assess all statistics in the manuscript, including the appropriateness of statistical tests used? Choose an item.

Quality of Written English

Please indicate the quality of language in the manuscript: Choose an item.

Declaration of Competing Interests

Please complete a declaration of competing interests, considering the following questions:

- Have you in the past five years received reimbursements, fees, funding, or salary from an organisation that may in any way gain or lose financially from the publication of this manuscript, either now or in the future?
- Do you hold any stocks or shares in an organisation that may in any way gain or lose financially from the publication of this manuscript, either now or in the future?
- Do you hold or are you currently applying for any patents relating to the content of the manuscript?

- Have you received reimbursements, fees, funding, or salary from an organization that holds or has applied for patents relating to the content of the manuscript?
- Do you have any other financial competing interests?
- Do you have any non-financial competing interests in relation to this paper?

If you can answer no to all of the above, write 'I declare that I have no competing interests' below. If your reply is yes to any, please give details below.

I declare that I have no competing interests

I agree to the open peer review policy of the journal. I understand that my name will be included on my report to the authors and, if the manuscript is accepted for publication, my named report including any attachments I upload will be posted on the website along with the authors' responses. I agree for my report to be made available under an Open Access Creative Commons CC-BY license (http://creativecommons.org/licenses/by/4.0/). I understand that any comments which I do not wish to be included in my named report can be included as confidential comments to the editors, which will not be published.

Choose an item.

To further support our reviewers, we have joined with Publons, where you can gain additional credit to further highlight your hard work (see: https://publons.com/journal/530/gigascience). On publication of this paper, your review will be automatically added to Publons, you can then choose whether or not to claim your Publons credit. I understand this statement.

Yes Choose an item.
