# Supplementary material for: Computational reproducibility of Jupyter notebooks from biomedical publications
Source: Gigascience. 2024 Jan 11;13:giad113. doi: 10.1093/gigascience/giad113 (PMC10783158; doi:10.1093/gigascience/giad113)
Supplement: giad113_Reviewer_2_Report_Revision_1 — Ryan Dale -- 9/11/2023 Reviewed [file giad113_reviewer_2_report_revision_1.pdf]

Reviewer Report

Title: Computational reproducibility of Jupyter notebooks from biomedical publications

Version: Revision 1 **Date:** 9/11/2023

Reviewer name: Ryan Dale

Reviewer Comments to Author:

Great to see the updated analysis; this is a valuable resource.
I at least wanted to try installing dependencies since that was a common failure mode of notebook reproducibility, but had some issues detailed below specifically for the Python 2.7 environments.
I did this on an x86_64 Ubuntu 22.04 docker image, after installing conda.
First, I added "source /conda/etc/profile.d/conda.sh" to the top of the script because otherwise "conda activate" wouldn't work inside the script. I also added "set -e" to the script so it would quit on any errors; otherwise if I let it run (defaulting to continuing on if there were any errors) it would be challenging to dig back through the very long output to manually look for any errors.
The installation script failed when trying to build the first "raw27" environment with this error:
Collecting widgetsnbextension~=3.6.5
Downloading widgetsnbextension-3.6.5-py2.py3-none-any.whl (1.6 MB)
|################################| 1.6 MB 62.9 MB/s
ERROR: Could not find a version that satisfies the requirement comm>=0.1.3 (from ipywidgets->jupyter->NotebookArchaeology==0.1) (from versions: 0.0.1)
ERROR: No matching distribution found for comm>=0.1.3 (from ipywidgets->jupyter->NotebookArchaeology==0.1)
So I commented out the building of that first environment, but the second one (py27) also failed:
Collecting cssselect
Downloading cssselect-1.1.0-py2.py3-none-any.whl (16 kB)
ERROR: Could not find a version that satisfies the requirement dnspython>=2.0.0 (from dkimpy; extra == "all"->yagmail[all]->NotebookArchaeology==0.1) (from versions: 1.11.0, 1.11.1, 1.12.0, 1.13.0, 1.14.0, 1.15.0, 1.16.0)
ERROR: No matching distribution found for dnspython>=2.0.0 (from dkimpy; extra == "all"->yagmail[all]->NotebookArchaeology==0.1)
Then I commented out lines so just the py37 env and onwards would build (hoping a more-recent python would work). It seemed to, except for a couple of envs I got a printed error that did still resulted in a 0 exit code. The error was this:
ERROR: pip's dependency resolver does not currently take into account all the packages that are installed. This behaviour is the source of the following dependency conflicts.
spyder 5.4.3 requires pyqt5<5.16, which is not installed.
spyder 5.4.3 requires pyqtwebengine<5.16, which is not installed.
python-lsp-black 1.2.1 requires black>=22.3.0, but you have black 0.0 which is incompatible.
So maybe these later ones are OK if they exited 0? I did not check to see if they did work for the analysis,

but wanted to at least point that out.

Methods

Are the methods appropriate to the aims of the study, are they well described, and are necessary controls included? Choose an item.

Conclusions

Are the conclusions adequately supported by the data shown? Choose an item.

Reporting Standards

Does the manuscript adhere to the journal's guidelines on minimum standards of reporting? Choose an item.

Choose an item.

Statistics

Are you able to assess all statistics in the manuscript, including the appropriateness of statistical tests used? Choose an item.

Quality of Written English

Please indicate the quality of language in the manuscript: Choose an item.

Declaration of Competing Interests

Please complete a declaration of competing interests, considering the following questions:

- Have you in the past five years received reimbursements, fees, funding, or salary from an organisation that may in any way gain or lose financially from the publication of this manuscript, either now or in the future?
- Do you hold any stocks or shares in an organisation that may in any way gain or lose financially from the publication of this manuscript, either now or in the future?
- Do you hold or are you currently applying for any patents relating to the content of the manuscript?
- Have you received reimbursements, fees, funding, or salary from an organization that holds or has applied for patents relating to the content of the manuscript?
- Do you have any other financial competing interests?
- Do you have any non-financial competing interests in relation to this paper?

If you can answer no to all of the above, write 'I declare that I have no competing interests' below. If your reply is yes to any, please give details below.

I declare that I have no competing interests

I agree to the open peer review policy of the journal. I understand that my name will be included on my report to the authors and, if the manuscript is accepted for publication, my named report including any attachments I upload will be posted on the website along with the authors' responses. I agree for my report to be made available under an Open Access Creative Commons CC-BY license (http://creativecommons.org/licenses/by/4.0/). I understand that any comments which I do not wish to be included in my named report can be included as confidential comments to the editors, which will not be published.

Choose an item.

To further support our reviewers, we have joined with Publons, where you can gain additional credit to further highlight your hard work (see: https://publons.com/journal/530/gigascience). On publication of this paper, your review will be automatically added to Publons, you can then choose whether or not to claim your Publons credit. I understand this statement.

Yes Choose an item.
